# Impact of *APOE* on amyloid and tau accumulation in argyrophilic grain disease and Alzheimer’s disease

**DOI:** 10.1186/s40478-024-01731-0

**Published:** 2024-02-09

**Authors:** Ana-Caroline Raulin, Sydney V. Doss, Michael G. Heckman, Emily C. Craver, Zonghua Li, Tadafumi C. Ikezu, Hiroaki Sekiya, Chia-Chen Liu, Yuka A. Martens, Cassandra L. Rosenberg, Lindsey A. Kuchenbecker, Michael DeTure, R. Ross Reichard, Aivi T. Nguyen, Eleni Constantopoulos, Rachel A. Larsen, Emmaline K. Kounaves, Melissa E. Murray, Dennis W. Dickson, Ronald C. Petersen, Guojun Bu, Takahisa Kanekiyo

**Affiliations:** 1 Department of Neuroscience, Mayo Clinic, Jacksonville, FL 32224 USA; 2 Division of Clinical Trials and Biostatistics, Department of Quantitative Health Sciences, Mayo Clinic, Jacksonville, FL 32224 USA; 3 Department of Laboratory Medicine and Pathology, Mayo Clinic, Rochester, MN 55905 USA; 4 Department of Neurology, Mayo Clinic, Rochester, MN 55905 USA; 5 Division of Life Science, The Hong Kong University of Science and Technology, Clear Water Bay, Hong Kong, China; 6 Present Address: SciNeuro Pharmaceuticals, Rockville, MD 20850 USA; 7 Present Address: Biogen, Cambridge, MA 02142 USA

**Keywords:** Amyloid-β, Alzheimer’s disease, Apolipoprotein E, Argyrophilic grain disease, MMSE, Tau

## Abstract

Alzheimer’s disease (AD), characterized by the deposition of amyloid-β (Aβ) in senile plaques and neurofibrillary tangles of phosphorylated tau (pTau), is increasingly recognized as a complex disease with multiple pathologies. AD sometimes pathologically overlaps with age-related tauopathies such as four repeat (4R)-tau predominant argyrophilic grain disease (AGD). While AGD is often detected with AD pathology, the contribution of *APOE4* to AGD risk is not clear despite its robust effects on AD pathogenesis. Specifically, how *APOE* genotype influences Aβ and tau pathology in co-occurring AGD and AD has not been fully understood. Using postmortem brain samples (N = 353) from a neuropathologically defined cohort comprising of cases with AD and/or AGD pathology built to best represent different *APOE* genotypes, we measured the amounts of major AD-related molecules, including Aβ40, Aβ42, apolipoprotein E (apoE), total tau (tTau), and pTau181, in the temporal cortex. The presence of tau lesions characteristic of AD (AD-tau) was correlated with cognitive decline based on Mini-Mental State Examination (MMSE) scores, while the presence of AGD tau lesions (AGD-tau) was not. Interestingly, while *APOE4* increased the risk of AD-tau pathology, it did not increase the risk of AGD-tau pathology. Although *APOE4* was significantly associated with higher levels of insoluble Aβ40, Aβ42, apoE, and pTau181, the *APOE4* effect was no longer detected in the presence of AGD-tau. We also found that co-occurrence of AGD with AD was associated with lower insoluble Aβ42 and pTau181 levels. Overall, our findings suggest that different patterns of Aβ, tau, and apoE accumulation mediate the development of AD-tau and AGD-tau pathology, which is affected by *APOE* genotype.

**Supplementary Information:**

The online version contains supplementary material available at 10.1186/s40478-024-01731-0.

## Introduction

**Fig. 1:**
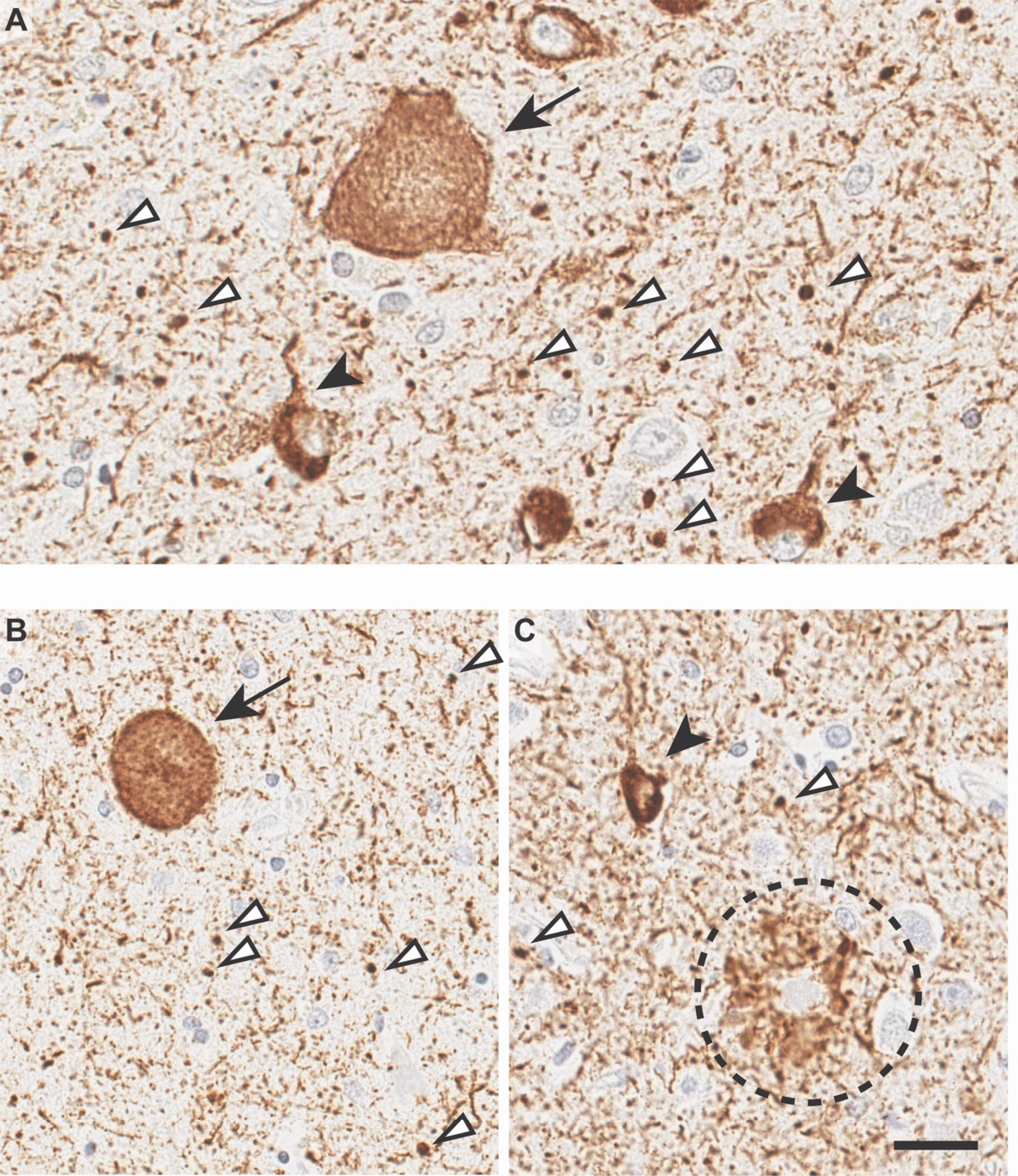
Representative images of AGD and of co-occurring AD with AGD. **A** Phosphorylated-tau immunohistochemistry (CP13) of the amygdala of a 94-year-old male patient with AGD. Arrows indicate balloon neurons, arrowheads indicate coiled bodies, and white triangle indicate grains. **B**, **C** Phosphorylated-tau immunohistochemistry (CP13) of the amygdala (**B**) and insula cortex (**C**) of a 91-year-old male patient with AGD and AD. Arrows indicate balloon neurons, arrowheads indicate coiled bodies, and white triangle indicate grains. Dashed circle shows neuritic plaque. Scale bar: 20 µm

Alzheimer’s disease (AD) is pathologically characterized by the extracellular deposition of amyloid-β (Aβ) in senile plaques and the intracellular accumulation of tau neurofibrillary tangles (NFT). However, proteinopathies caused by α-synuclein and by TDP-43 as well as vascular lesions are frequently observed in AD brains [6]. The presence of these additional neuropathological changes is predicted to impact AD phenotypes and progression [3]. Argyrophilic grain disease (AGD) is a common sporadic age-related primary tauopathy, which often coexists with AD (Fig. 1). AGD is defined by the presence of spindle- or comma-shaped argyrophilic grains in the neuropil of several brain regions, including the entorhinal cortex, hippocampus, and amygdala [6, 36]. Argyrophilic grains are neurofibrillary lesions enriched in 4-repeat (4R) tau, in contrast to AD neurofibrillary tangles composed of both 3R and 4R tau aggregates [37]. AGD is detected in approximately 5% of dementia cases [35, 36]. Intriguingly, a neuropathological study has reported that AD patients with AGD have lower scores of amyloid and tau pathologies than those without AGD [35]. While *APOE* gene coding apolipoprotein E (apoE) is the most significant genetic modifier for AD risk, *APOE* is also significantly tied with the occurrence of AGD. Among the three major *APOE* alleles, *APOE2* has been demonstrated to increase the risk for AGD onset [8], which is in contrast to its protective effect in AD [17]. Although *APOE4* is associated with a dose-dependent risk for AD with a 15-fold increased risk in *APOE4* homozygotes [26], a lack of relationship between *APOE4* and AGD onset has also been reported [36, 38].

In this study, using a large, neuropathologically defined cohort of postmortem brain samples with different *APOE* genotypes (N = 353), we biochemically investigated how *APOE* genotype is associated with the levels of major AD-related molecules, including Aβ40, Aβ42, total tau (tTau), phosphorylated tau 181 (pTau181), and apoE, in the presence of AGD and/or AD pathologies. Our findings revealed that the presence of neuropathologically defined lesions characteristic of AGD-tau pathology is associated with lower levels of Aβ40 and p-tau181 in mixed AD cases, with the association between *APOE4* and the AD-related molecules levels being less pronounced in the presence of AGD-tau.

## Materials and methods

### Human neuropathological assessment

Postmortem brain tissue from non-Hispanic White donors was obtained from a group of 437 autopsied study participants identified from the Alzheimer’s Disease Research Center (P30 AG062677) and Mayo Clinic Study of Aging (U01 AG006786) with inclusion criteria requiring antemortem diagnosis within one year of death of clinical continuum of AD (i.e., normal, mild cognitive impairment, probable/possible AD), frozen tissue availability, tissue blocks availability, and lack of primary tauopathy (e.g. progressive supranuclear palsy, corticobasal degeneration, Pick’s disease, globular glial tauopathy). Of the 437 identified, a total of 353 autopsied non-Hispanic White donors were selected to best span the different *APOE* genotypes. Standard genotyping methods on blood samples was used to determine *APOE* allele status (ε2, ε3, ε4) [12]. The *APOE2* group includes *APOE* ε2/ε2 (N = 1) and *APOE* ε2/ε3 (N = 45). The *APOE3* group consists of *APOE* ε3/ε3 (N = 162) genotype. The *APOE4* group includes *APOE* ε3/ε4 (N = 114) and *APOE* ε4/ε4 (N = 43). Cases with *APOE* ε2/ε4 *(*N = 22) were excluded because their limited representation in our dataset raises concerns about statistical robustness. Additionally, MMSE score was available in 133 subjects. Neuropathological examinations of brain tissue were performed in accordance with standardized protocols approved by Mayo Clinic Institutional Review board, as previously described [23]. These include neuropathologic evaluation using immunohistochemistry for antibodies against Aβ (Clone 6F/3D, DAKO), tau (AT8, ThermoFisher), TDP-43 (p409/410; Cosmo Bio), and α-synuclein (LB 509, Abcam). The diagnosis of AD neuropathologic change (ADNC) was conducted using the 2012 NIA-AA criteria [21], a well-established neuropathologic consensus criteria which include examination of AD-related pathologies such as Thal phase for Aβ plaques [34], and Braak NFT stage [1, 2]. Neuritic plaque semiquantitative scores were employed in our analyses: 0 = None; 1 = Sparse; 2 = Moderate; 3 = Frequent [21].

AGD was first screened using tau (AT8) immunohistochemistry in the amygdala, and later confirmed with 4R tau isoform (RD4, clone 1E1/A6, Millipore) immunohistochemistry and Bielschowsky silver stain, in conjunction with histomorphologic findings of ballooned neurons and other pertinent features on H&E-stained sections. Sections from the hippocampus, amygdala, and anterior cingulate are frequently screened and utilized for the diagnosis of AGD (Fig. 1). For analyses purposes, we have defined the following criteria: “AD-tau negative” (AD-tau = 0) corresponds to Braak stage < 4; “AD-tau positive” (AD-tau = 1) corresponds to Braak stage ≥ 4 [1]; “AGD-tau negative” (AGD-tau = 0) indicates the absence of AGD comorbidity with AD; “AGD-tau positive” (AGD-tau = 1) indicates the presence of AGD comorbidity with AD.

### Sample preparation

Dissected tissues from the temporal cortex (100 mg) were pulverized and subjected to three-step extraction to isolate proteins according to their solubility in Tris-buffered saline (TBS), detergent-containing TBS, or formic acid (FA), as detailed previously [18, 33]. Briefly, samples were homogenized in 10 volumes (w/v) of ice-cold TBS supplemented with a protease inhibitor cocktail (Roche Diagnostics) and a phosphatase inhibitor (Roche Diagnostics) by Polytron homogenizer (KINEMATICA). Brain homogenates were centrifuged at 100,000 × g for 60 min at 4 ºC. The supernatant (soluble fraction) was collected, and the residual pellet was resuspended in 10 volumes of TBS containing 1% Triton-X (TBSX), supplemented with protease and phosphatase inhibitors. Following sonication, samples were incubated at 4 °C for 30 min with end-over-end agitation and centrifuged as described above. The resulting supernatant (detergent-soluble fraction) was retrieved, and the resulting pellet was re-solubilized in 70% FA. Samples were sonicated, incubated overnight at 4 °C with end-over-end agitation, and centrifuged as above. The final supernatant (insoluble fraction) was recovered and neutralized 20-fold with 1 M Tris-buffer (pH 11). All collected fractions were aliquoted and stored at − 80 °C until use.

### Quantification of AD-related proteins

Amounts of Aβ40, Aβ42, apoE, tTau, and pTau181 in soluble, detergent-soluble, and insoluble fractions were determined by enzyme-linked immunosorbent assay (ELISA). Aβ40 and Aβ42 were measured using sandwich ELISA with antibodies produced in-house at Mayo Clinic, as previously described [5]. Briefly, end-specific monoclonal antibodies (13.1.1 for Aβ40 and 2.1.3 for Aβ42) were used as capture antibodies, and a horseradish peroxidase (HRP)-conjugated monoclonal antibody (Ab5-HRP) was used for detection. ApoE were quantified by sandwich ELISA with a polyclonal antibody directed against apoE (AB947, Millipore) used as capture antibody and a biotin-conjugated polyclonal anti-apoE antibody (K74180B, Meridian Life Sciences) used as detection antibody. An HRP-streptavidin conjugate was used to bind the biotinylated detection antibody [18]. For sandwich ELISA for tTau, monoclonal tau antibody (HT7; ThermoFisher Scientific) and a biotin-conjugated monoclonal anti-tau antibody (BT2; ThermoFisher Scientific) were utilized as capture and detection antibodies, respectively. An HRP-streptavidin conjugate was added to interact with the biotinylated detection antibody [18]. Color development for in-house sandwich ELISAs was initiated by addition of 3,3′,5,5′- tetramethylbenzidine (TMB) substrate, and the reaction was stopped with 1 M sulfuric acid. Absorbance was measured at 450 nm using a Synergy HT microplate reader (BioTek). Target protein levels were calculated using respective standard curves. For pTau181, a commercially available ELISA kit (ThermoFisher Scientific) was used according to the manufacturer’s instructions. All protein levels measured by ELISA were normalized against total protein concentration quantified using Pierce Detergent Compatible Bradford assay kit (ThermoFisher Scientific). Due to limits of detection in our ELISA assays, analytes could not be measured for a small amount (< 15%) of brain lysates samples.

### Statistical analysis

Comparisons of characteristics according to *APOE* genotype group, and also according to combination of AD-tau pathology and AGD-tau pathology, were made using a Kruskal–Wallis rank sum test or a Wilcoxon rank sum test (continuous and ordinal variables) or Fisher’s exact test (categorical variables). Associations of presence of *APOE2* or *APOE4* with amyloid score, AD-tau, and AGD-tau were evaluated using unadjusted and age/sex-adjusted proportional odds logistic regression models (amyloid score) and binary logistic regression models (AD-tau and AGD-tau); p-values < 0.0167 were considered as statistically significant after applying a Bonferroni correction for multiple testing for the three outcome measures that were assessed.

Associations of demographic and neuropathological characteristics with MMSE score and AD-related molecules were evaluated using linear regression models. First, unadjusted models were assessed. Second, models were adjusted for age and sex only. Finally, a full multivariable analysis was performed adjusting for age, sex, and also any other variable with a *P*-value < 0.05 in unadjusted analysis for the given outcome (MMSE score or the AD-related molecule). *P*-values < 0.005 were considered as statistically significant after applying a Bonferroni correction for multiple testing for the 10 characteristics that were assessed for association with each outcome. AD-related molecules were examined on the square root, cube root, or natural logarithm scales in all regression analysis due to the presence of skewed distributions. Interactions with AD-tau and AGD-tau were also assessed in age/sex-adjusted linear regression models, where *P*-values < 0.0056 were considered as significant after Bonferroni correction.

Comparisons of AD-related molecules between *APOE* groups (*APOE2* vs. *APOE3* and *APOE4* vs. *APOE3*) were made using unadjusted and age/sex-adjusted linear regression models. AD-related molecules were examined on the square root, cube root, or natural logarithm scales in all regression analysis due to the presence of skewed distributions; interactions with combination of AD-tau and AGD-tau were also assessed. Associations of MMSE score with AD-related molecules were also examined using unadjusted and age/sex-adjusted linear regression models. *P*-values < 0.01 were considered significant after applying a Bonferroni correction separately for each fraction. All statistical tests were two-sided. Statistical analysis was performed using R Statistical Software (version 4.1.2; R Foundation for Statistical Computing, Vienna, Austria).

## Results

### *APOE* genotype influences neuropathology in the elderly

We investigated postmortem brain samples from our study cohort consisting of 353 subjects (174 males and 179 females) chosen to best represent different *APOE* genotypes, with a mean age at death of 89 years in the *APOE2* group (range: 69–101 years), 89 years in the *APOE3* group (range: 59–100 years), and 84 years in the *APOE4* group (range: 54–103 years). When comparing demographic and select neuropathological characteristics between the three *APOE* genotype groups (Table 1), we found that *APOE* genotype predominantly influenced both amyloid and tau pathology, with more severe scores of Braak stage and Thal phase detected in the *APOE4* group. In more detailed analysis of neuritic plaque score, AD tau pathology, and AGD-tau pathology (Table 2), neuritic plaque score was significantly (P < 0.0167 considered significant after multiple testing correction) lower in the presence of *APOE2* (OR = 0.49, *p* = 0.015), but higher in the presence of *APOE4* (OR = 4.86, *p* < 0.001) when adjusting for age and sex. Additionally, the presence of *APOE4* was associated with a higher likelihood of AD-tau pathology (OR = 6.34, *p* < 0.001), while although not quite significant, AGD-tau prevalence was lower in the presence of *APOE4* (OR = 0.49, *p* = 0.041). *APOE2* was associated with a significantly lower odds than *APOE4* of AD-tau occurrence (OR = 0.40, *p* = 0.007); however, it was not associated with AGD-tau occurrence (OR = 1.89, *p* = 0.11).

**Table 1:** Subject characteristics according to *APOE* genotype

Variable	*APOE2* (N = 45)		*APOE3* (N = 156)		*APOE4* (N = 152)		*P*-value
	N	Median (minimum, maximum) or No. (%) of patients	N	Median (minimum, maximum) or No. (%) of patients	N	Median (minimum, maximum) or No. (%) of patients	
Age at death (years)	45	89 (69, 101)	156	89 (59, 100)	152	84 (54, 103)	< 0.001
Sex (Male)	45	19 (42.2%)	156	71 (45.5%)	152	84 (55.3%)	0.14
MMSE score	19	25 (17, 29)	83	27 (18, 30)	31	27 (18, 29)	0.083
Braak stage	45		154		148		< 0.001
0		0 (0.0%)		4 (2.6%)		2 (1.4%)	
1		5 (11.1%)		17 (11.0%)		6 (4.1%)	
2		13 (28.9%)		40 (26.0%)		8 (5.4%)	
3		11 (24.4%)		32 (20.8%)		12 (8.1%)	
4		6 (13.3%)		34 (22.1%)		24 (16.2%)	
5		4 (8.9%)		21 (13.6%)		38 (25.7%)	
6		6 (13.3%)		6 (3.9%)		58 (39.2%)	
Thal phase	28		105		59		< 0.001
0		9 (32.1%)		22 (21.0%)		5 (8.5%)	
1		5 (17.9%)		26 (24.8%)		3 (5.1%)	
2		2 (7.1%)		12 (11.4%)		5 (8.5%)	
3		6 (21.4%)		30 (28.6%)		10 (16.9%)	
4		2 (7.1%)		5 (4.8%)		6 (10.2%)	
5		4 (14.3%)		10 (9.5%)		30 (50.8%)	
VaD	45	20 (44.4%)	156	62 (39.7%)	152	41 (27.0%)	0.019
CAA	45	5 (11.1%)	156	6 (3.8%)	152	13 (8.6%)	0.088
TDP-43	45	2 (4.4%)	156	6 (3.8%)	152	19 (12.5%)	0.012
Synuclein	45	9 (20.0%)	156	25 (16.0%)	152	50 (32.9%)	0.002

*P*-values result from a Kruskal–Wallis rank sum test (continuous and ordinal variables) or Fisher’s exact test (categorical variables)

**Table 2:** Associations of *APOE2* and *APOE4* with neuritic plaque score, AD-tau, and AGD-tau

Variable	*APOE2* present		*APOE2* absent		Unadjusted analysis		Adjusting for age and sex	
	N	Median (minimum, maximum) or No. (%) of subjects	N	Median (minimum, maximum) or No. (%) of subjects	Estimate (95% CI)	*P*-value	Estimate (95% CI)	*P*-value
Neuritic plaque score	45		308		**0.44 (0.25, 0.78)**	**0.005**	**0.49 (0.28, 0.87)**	**0.015**
0		20 (44.4%)		60 (19.5%)				
1		4 (8.9%)		62 (20.1%)				
2		10 (22.2%)		76 (24.7%)				
3		11 (24.4%)		110 (35.7%)				
AD-tau	45	16 (35.6%)	302	181 (59.9%)	**0.37 (0.19, 0.70)**	**0.003**	**0.40 (0.20, 0.78)**	**0.007**
AGD-tau	45	11 (24.4%)	308	40 (13.0%)	2.17 (0.98, 4.51)	0.045	1.89 (0.84, 4.00)	0.11

Variable	*APOE4* present		*APOE4* absent		Unadjusted analysis		Adjusting for age and sex	
	N	Median (minimum, maximum) or No. (%) of subjects	N	Median (minimum, maximum) or No. (%) of subjects	Estimate (95% CI)	*P*-value	Estimate (95% CI)	*P*-value
Neuritic plaque score	152		201		**5.31 (3.51, 8.05)**	** < 0.001**	**4.86 (3.17, 7.46)**	** < 0.001**
0		7 (4.6%)		73 (36.3%)				
1		26 (17.1%)		40 (19.9%)				
2		37 (24.3%)		49 (24.4%)				
3		82 (53.9%)		39 (19.4%)				
AD-tau	148	120 (81.1%)	199	77 (38.7%)	**6.79 (4.16, 11.36)**	** < 0.001**	**6.34 (3.83, 10.76)**	** < 0.001**
AGD-tau	152	13 (8.6%)	201	38 (18.9%)	**0.40 (0.20, 0.76)**	**0.007**	0.49 (0.24, 0.95)	0.041

*CI* Confidence interval. For neuritic plaque score, odds ratios, 95% CIs, and p-values result from proportional odds logistic regression models; odds ratios are interpreted as the multiplicative increase in the odds of a higher neuritic plaque score corresponding to presence of *APOE2* or *APOE4*. For AD-tau and AGD-tau, odds ratios, 95% CIs, and p-value result from binary logistic regression models; odds ratios are interpreted as the multiplicative increase in the odds of the given outcome (AD-tau or AGD-tau) corresponding to presence of *APOE2* or *APOE4*. P-values < 0.0167 were considered as statistically significant after applying a Bonferroni correction for multiple testing; significant findings are shown in bold

### AD-tau pathology is associated with cognitive impairment

Among the cases available for MMSE score in the cohort, we investigated the association between demographic and neuropathological measures and MMSE scores (Additional file 1: Table S1). After correcting for multiple testing (*P* < 0.005 considered significant), significant negative associations with MMSE scores were observed for both older age (β = − 1.33, *p* < 0.001) and the presence of AD-tau lesions (β = − 2.11, *p* < 0.001) in analysis that was adjusted for age and sex. Furthermore, findings remained significant in full multivariable analysis when additionally adjusting for all variables with a p-value < 0.05 in unadjusted analysis (AD-tau) for both age (β = − 1.07, *p* = 0.004) and the presence of AD-tau (β = − 2.03, *p* < 0.001). When potential interactive effects of presence of AD-tau and AGD-tau with demographic/neuropathological characteristics were examined regarding associations with MMSE score, with adjustment for age and sex, no significant interactions were identified after correcting for multiple testing (Additional file 1: Table S2). Of note, the presence of AD-tau lesions was significantly associated with lower MMSE scores only in the absence of AGD-tau pathology (β = − 2.56, *p* < 0.001), with a weaker and non-significant association for subjects with AGD-tau pathology (β = − 0.24, *p* = 0.82); however, this interaction did not reach statistical significance (*p* = 0.042).

### *APOE* genotype influences the levels of AD-related molecules in the temporal cortex

We compared the brain levels of AD-related molecules including Aβ40, Aβ42, apoE, tTau, and pTau181 in the soluble (TBS), detergent-soluble (TBSX), and insoluble (FA) fractions of brain lysate between *APOE* genotype groups (Table 3, Additional file 1: Table S3). Following adjustment for age and sex and after correcting for multiple testing (P < 0.01 considered as significant), we found significantly higher soluble apoE levels in the *APOE2* group compared to the *APOE3* group (β = 0.61 *p* < 0.001). We also found numerous differences between *APOE4* and *APOE3* groups. Aβ40 levels were higher in the *APOE4* group than in the *APOE3* group in the soluble, detergent-soluble, and insoluble fractions (TBS: β = 0.82, *p* = 0.005; TBSX: β = 0.99, *p* < 0.001; FA: β = 14.04, *p* < 0.001). Aβ42 levels were also higher in all three fractions in the *APOE4* group compared to the *APOE3* group (TBS: β = 2.29, *p* < 0.001; TBSX: β = 2.51, *p* < 0.001; FA: β = 24.96, *p* < 0.001). Compared to *APOE3*, *APOE4* was associated with increased levels of insoluble apoE (FA: β = 1.24, *p* < 0.001) and insoluble pTau181 (FA: β = 0.72, *p* < 0.001), and decreased detergent-soluble tTau levels (TBSX: β = -0.18, *p* = 0.002). Additionally, among the measured analytes, there was only a positive association between MMSE score and insoluble tTau levels after adjusting for age and sex (FA: β = 0.274, *p* = 0.007) (Additional file 1: Table S4).

**Table 3:** Comparisons of AD-related molecules between *APOE* genotype groups

	N	β (95% CI)	*P*-value
*APOE2* vs. *APOE3* (reference)		*APOE2* (N = 45) vs. *APOE3* (N = 156)	
Aβ40-TBS	174	0.36 (− 0.36, 1.08)	0.32
Aβ40-TBSX	188	− 0.26 (− 0.78, 0.26)	0.33
Aβ40-FA	188	1.12 (− 2.61, 4.86)	0.55
Aβ42-TBS	186	0.31 (− 0.84, 1.46)	0.59
Aβ42-TBSX	192	− 0.98 (− 2.02, 0.06)	0.064
Aβ42-FA	197	− 1.07 (− 12.12, 9.99)	0.85
apoE-TBS	196	**0.61 (0.29, 0.93)**	** < 0.001**
apoE-TBSX	194	− 0.11 (− 0.26, 0.04)	0.14
apoE-FA	197	0.03 (− 0.46, 0.52)	0.91
tTau-TBS	176	− 2.44 (− 7.41, 2.53)	0.33
tTau-TBSX	194	− 0.03 (− 0.19, 0.12)	0.66
tTau-FA	201	0.14 (− 0.02, 0.29)	0.079
pTau181-TBS	194	− 0.05 (− 0.23, 0.13)	0.56
pTau181-TBSX	194	− 0.08 (− 0.21, 0.04)	0.18
pTau181-FA	192	− 0.01 (− 0.27, 0.25)	0.94
*APOE4* vs. *APOE3* (reference)		*APOE4* (N = 152) vs. *APOE3* (N = 156)	
Aβ40-TBS	267	**0.82 (0.25, 1.39)**	**0.005**
Aβ40-TBSX	292	**0.99 (0.51, 1.48)**	** < 0.001**
Aβ40-FA	290	**14.04 (9.94, 18.15)**	** < 0.001**
Aβ42-TBS	289	**2.29 (1.63, 2.96)**	** < 0.001**
Aβ42-TBSX	293	**2.51 (1.88, 3.14)**	** < 0.001**
Aβ42-FA	303	**24.96 (18.17, 31.75)**	** < 0.001**
apoE-TBS	303	− 0.29 (− 0.51, − 0.06)	0.012
apoE-TBSX	296	0.13 (0.02, 0.23)	0.016
apoE-FA	302	**1.24 (0.85, 1.64)**	** < 0.001**
tTau-TBS	278	− 1.62 (− 5.16, 1.91)	0.37
tTau-TBSX	296	− **0.18 (**− **0.30,** − **0.07)**	**0.002**
tTau-FA	308	− 0.13 (− 0.24, − 0.03)	0.013
pTau181-TBS	297	− 0.14 (− 0.26, − 0.03)	0.017
pTau181-TBSX	295	− 0.06 (− 0.15, 0.02)	0.14
pTau181-FA	296	**0.72 (0.49, 0.95)**	** < 0.001**

β Regression coefficient; *CI* Confidence interval. β coefficients, 95% CIs, and p-values result from linear regression models that were adjusted for age and sex. β values are interpreted as the difference in means of the given AD-related molecule on the square root (tTau-TBS), cube root (apoE-TBSX, tTau-TBSX, tTau-FA), or natural logarithm scale (Aβ40-TBS, Aβ40-TBSX, Aβ40-FA, Aβ42-TBS, Aβ42-TBSX, Aβ42-FA, apoE-TBS, apoE-FA, pTau181-TBS, pTau181-TBSX, pTau181-FA) in comparison to the *APOE3* group. *P*-values < 0.01 were considered as statistically significant after applying a Bonferroni correction for multiple testing separately for each fraction and each pair-wise comparison between *APOE* groups; significant findings are shown in bold

### Neuropathological measures are associated with the levels of AD-related molecules in the temporal cortex

Regression analyses were conducted to examine the independent associations of neuropathological measures for VaD, CAA, amyloid score, TDP-43, synuclein, AD-tau, and AGD-tau with AD-related molecules (Table 4 [FA], and Additional file 1: Tables S5 [TBS] and S6 [TBSX]). In full multivariable analysis adjusting for age, sex, and any other measure that was associated with the given AD-related molecule with P < 0.05 in unadjusted analysis, neuritic plaque score was significantly associated (*p* < 0.005 considered as significant) with increased levels of soluble, detergent-soluble, and insoluble Aβ42 (TBS: β = 1.23, *p* < 0.001; TBSX: β = 1.33, *p* < 0.001; FA: β = 13.03, *p* < 0.001), insoluble apoE (FA: β = 0.65, *p* < 0.001) and insoluble pTau-181 (FA: β = 0.31, *p* < 0.001). The presence of CAA was also associated with increased levels of soluble, detergent-soluble, and insoluble Aβ40 (TBS: β = 2.30, *p* < 0.001; TBSX: β = 1.41, p = 0.001; FA: β = 20.94, *p* < 0.001) in full multivariable analysis. AD-tau pathology was positively associated with the levels of insoluble Aβ42 (FA: β = 12.27, *p* < 0.001) and pTau181 (FA: β = 0.41, *p* = 0.001), as well as negatively associated with levels of detergent-soluble tTau (TBSX: β = -0.23, *p* < 0.001), in full multivariable analysis. There were no associations that withstood correction for multiple testing between AD-related molecules and VaD, TDP-43, synuclein, or AGD-tau in full multivariable analysis.

**Table 4:** Associations of neuropathological measures with AD-related molecules (FA)

Variable	N	Unadjusted analysis		Adjusting for age and sex		Full multivariable analysis	
		β (95% CI)	P-value	β (95% CI)	P-value	β (95% CI)	P-value
Association with Aβ40-FA							
Age	333	− 2.58 (− 5.02, − 0.14)	0.038	− 2.45 (− 4.95, 0.06)	0.056	0.60 (− 1.64, 2.83)	0.60
Sex	333	1.79 (− 2.06, 5.64)	0.36	0.91 (− 3.03, 4.85)	0.65	− 0.62 (− 4.07, 2.83)	0.72
*APOE4*	333	**14.02 (10.43, 17.60)**	** < 0.001**	**13.87 (10.15, 17.59)**	** < 0.001**	**8.78 (4.93, 12.62)**	** < 0.001**
VaD	333	− 1.43 (− 5.46, 2.60)	0.49	− 0.98 (− 5.06, 3.11)	0.64	− 0.41 (− 3.98, 3.16)	0.82
CAA	333	**22.92 (15.89, 29.96)**	** < 0.001**	**22.76 (15.67, 29.84)**	** < 0.001**	**20.94 (14.49, 27.40)**	** < 0.001**
Neuritic plaque score	333	**6.05 (4.53, 7.57)**	** < 0.001**	**5.97 (4.43, 7.52)**	** < 0.001**	2.67 (0.62, 4.71)	0.011
TDP-43	333	0.44 (− 6.88, 7.76)	0.91	0.82 (− 6.51, 8.15)	0.83	− 3.81 (− 10.17, 2.55)	0.24
Synuclein	333	5.54 (1.08, 9.99)	0.015	4.80 (0.27, 9.33)	0.038	0.17 (− 3.85, 4.19)	0.93
AD-tau	327	**12.48 (8.77, 16.19)**	** < 0.001**	**12.23 (8.46, 16.01)**	** < 0.001**	4.39 (− 0.28, 9.06)	0.065
AGD-tau	333	− 7.71 (− 13.13, − 2.28)	0.006	− 7.02 (− 12.52, − 1.52)	0.012	− 2.40 (− 7.28, 2.49)	0.33
Association with Aβ42-FA							
Age	348	− 4.10 (− 8.31, 0.12)	0.057	− 4.15 (− 8.50, 0.20)	0.062	2.71 (− 0.70, 6.11)	0.12
Sex	348	1.25 (− 5.53, 8.03)	0.72	− 0.34 (− 7.30, 6.61)	0.92	1.47 (− 3.80, 6.73)	0.58
*APOE4*	348	**25.05 (18.75, 31.36)**	** < 0.001**	**25.30 (18.69, 31.90)**	** < 0.001**	7.60 (1.68, 13.53)	0.012
VaD	348	− 0.66 (− 7.78, 6.45)	0.86	0.46 (− 6.77, 7.68)	0.90	3.48 (− 1.99, 8.95)	0.21
CAA	348	**20.19 (6.72, 33.66)**	**0.003**	**19.91 (6.34, 33.47)**	**0.004**	14.11 (3.86, 24.35)	0.007
Neuritic plaque score	348	**18.06 (15.85, 20.27)**	** < 0.001**	**18.31 (16.05, 20.57)**	** < 0.001**	**13.03 (9.93, 16.13)**	** < 0.001**
TDP-43	348	11.83 (− 0.77, 24.44)	0.066	12.04 (− 0.60, 24.67)	0.062	0.18 (− 9.42, 9.77)	0.97
Synuclein	348	11.02 (3.15, 18.88)	0.006	9.97 (1.94, 18.00)	0.015	− 1.23 (− 7.39, 4.94)	0.70
AD-tau	342	**35.46 (29.68, 41.25)**	** < 0.001**	**35.42 (29.50, 41.34)**	** < 0.001**	**12.27 (5.23, 19.31)**	** < 0.001**
AGD-tau	348	− **19.91 (**− **29.26,** − **10.56)**	** < 0.001**	− **19.18 (**− **28.66,** − **9.70)**	** < 0.001**	− 3.81 (− 11.20, 3.58)	0.31
Association with apoE-FA							
Age	346	− **0.53 (**− **0.76,** − **0.29)**	** < 0.001**	− **0.51 (**− **0.75,** − **0.27)**	** < 0.001**	− 0.25 (− 0.48, − 0.02)	0.031
Sex	346	0.30 (− 0.08, 0.69)	0.12	0.12 (− 0.26, 0.51)	0.53	0.14 (− 0.20, 0.49)	0.42
*APOE4*	346	**1.39 (1.03, 1.76)**	** < 0.001**	**1.25 (0.88, 1.63)**	** < 0.001**	**0.79 (0.39, 1.18)**	** < 0.001**
VaD	346	− 0.36 (− 0.76, 0.05)	0.082	− 0.24 (− 0.64, 0.17)	0.25	− 0.09 (− 0.45, 0.28)	0.63
CAA	346	**1.26 (0.51, 2.02)**	**0.001**	**1.17 (0.43, 1.91)**	**0.002**	0.82 (0.15, 1.49)	0.016
Neuritic plaque score	346	**0.74 (0.59, 0.89)**	** < 0.001**	**0.70 (0.55, 0.85)**	** < 0.001**	**0.65 (0.44, 0.85)**	** < 0.001**
TDP-43	346	0.43 (− 0.30, 1.17)	0.25	0.51 (− 0.21, 1.23)	0.16	0.04 (− 0.61, 0.69)	0.91
Synuclein	346	0.55 (0.09, 1.00)	0.018	0.38 (− 0.07, 0.83)	0.10	− 0.15 (− 0.56, 0.26)	0.47
AD-tau	340	**1.08 (0.70, 1.46)**	** < 0.001**	**0.96 (0.58, 1.34)**	** < 0.001**	− 0.30 (− 0.77, 0.16)	0.20
AGD-tau	346	− 0.64 (− 1.19, − 0.10)	0.021	− 0.48 (− 1.03, 0.06)	0.080	0.04 (− 0.45, 0.54)	0.86
Association with tTau-FA							
Age	353	0.03 (− 0.03, 0.09)	0.29	0.03 (− 0.03, 0.09)	0.37	0.00 (-0.07, 0.06)	0.92
Sex	353	− 0.04 (− 0.14, 0.06)	0.41	− 0.03 (− 0.13, 0.07)	0.56	− 0.03 (− 0.13, 0.07)	0.52
*APOE4*	353	− **0.16 (**− **0.26,** − **0.06)**	**0.001**	− **0.16 (**− **0.26,** − **0.05)**	**0.003**	− 0.11 (− 0.22, 0.00)	0.059
VaD	353	0.01 (− 0.09, 0.12)	0.79	0.01 (− 0.10, 0.11)	0.88	0.02 (− 0.09, 0.12)	0.74
CAA	353	− 0.06 (− 0.26, 0.13)	0.52	− 0.05 (− 0.25, 0.14)	0.60	− 0.05 (− 0.25, 0.14)	0.61
Neuritic plaque score	353	− 0.04 (− 0.09, − 0.00)	0.041	− 0.04 (− 0.09, 0.00)	0.055	0.01 (− 0.05, 0.06)	0.82
TDP-43	353	− 0.13 (− 0.32, 0.05)	0.15	− 0.14 (− 0.33, 0.04)	0.13	− 0.08 (− 0.27, 0.10)	0.37
Synuclein	353	− 0.11 (− 0.22, 0.01)	0.069	− 0.10 (− 0.21, 0.02)	0.11	− 0.08 (− 0.20, 0.04)	0.21
AD-tau	347	− **0.15 (**− **0.25,** − **0.05)**	**0.003**	− **0.15 (**− **0.25,** − **0.05)**	**0.004**	− 0.11 (− 0.25, 0.02)	0.098
AGD-tau	353	0.08 (− 0.06, 0.22)	0.27	0.07 (− 0.08, 0.21)	0.36	0.02 (− 0.12, 0.17)	0.74
Association with pTau181-FA							
Age	340	− **0.39 (**− **0.52,** − **0.25)**	** < 0.001**	− **0.40 (**− **0.54,** − **0.26)**	** < 0.001**	− **0.22 (**− **0.34,** − **0.10)**	** < 0.001**
Sex	340	0.06 (− 0.17, 0.29)	0.62	− 0.10 (− 0.32, 0.13)	0.39	− 0.07 (− 0.26, 0.12)	0.45
*APOE4*	340	**0.85 (0.64, 1.07)**	** < 0.001**	**0.74 (0.53, 0.96)**	** < 0.001**	0.28 (0.07, 0.49)	0.010
VaD	340	− 0.08 (− 0.32, 0.15)	0.49	0.04 (− 0.19, 0.27)	0.73	0.10 (− 0.10, 0.29)	0.34
CAA	340	0.46 (0.02, 0.90)	0.041	0.44 (0.01, 0.87)	0.044	0.29 (− 0.06, 0.65)	0.10
Neuritic plaque score	340	**0.52 (0.44, 0.61)**	** < 0.001**	**0.49 (0.41, 0.57)**	** < 0.001**	**0.31 (0.20, 0.42)**	** < 0.001**
TDP-43	340	0.39 (− 0.04, 0.83)	0.077	0.41 (− 0.01, 0.83)	0.056	0.03 (− 0.32, 0.38)	0.87
Synuclein	340	0.35 (0.09, 0.62)	0.009	0.22 (− 0.04, 0.49)	0.092	− 0.08 (− 0.30, 0.14)	0.50
AD-tau	334	**1.09 (0.89, 1.28)**	** < 0.001**	**1.00 (0.81, 1.20)**	** < 0.001**	**0.41 (0.16, 0.66)**	**0.002**
AGD-tau	340	− **0.57 (**− **0.90,** − **0.24)**	** < 0.001**	− **0.48 (**− **0.80,** − **0.17)**	**0.003**	− 0.08 (− 0.35, 0.19)	0.57

*β* regression coefficient; *CI* Confidence interval. β values, 95% CIs, and p-values result from linear regression models. β values are interpreted as the change in mean AD-related molecule on the cube root (Aβ40-FA, Aβ42-FA, apoE-FA, pTau181-FA) or natural logarithm scale (tTau-FA) corresponding to each 10-year increase in age, male sex, presence of *APOE4*, presence of VaD, presence of CAA, 1 unit increase in neuritic plaque score, presence of TDP-43 pathology, presence of synucleinopathy, presence of AD-tau or presence of AGD-tau. Full multivariable models were adjusted for age, sex, and all variables with an association *P*-value < 0.05 in the unadjusted analysis for the given AD-related molecule. *P*-values < 0.005 were considered as statistically significant after applying a Bonferroni correction for multiple testing separately for each AD-related molecule; significant findings are shown in bold

**Fig. 2:**
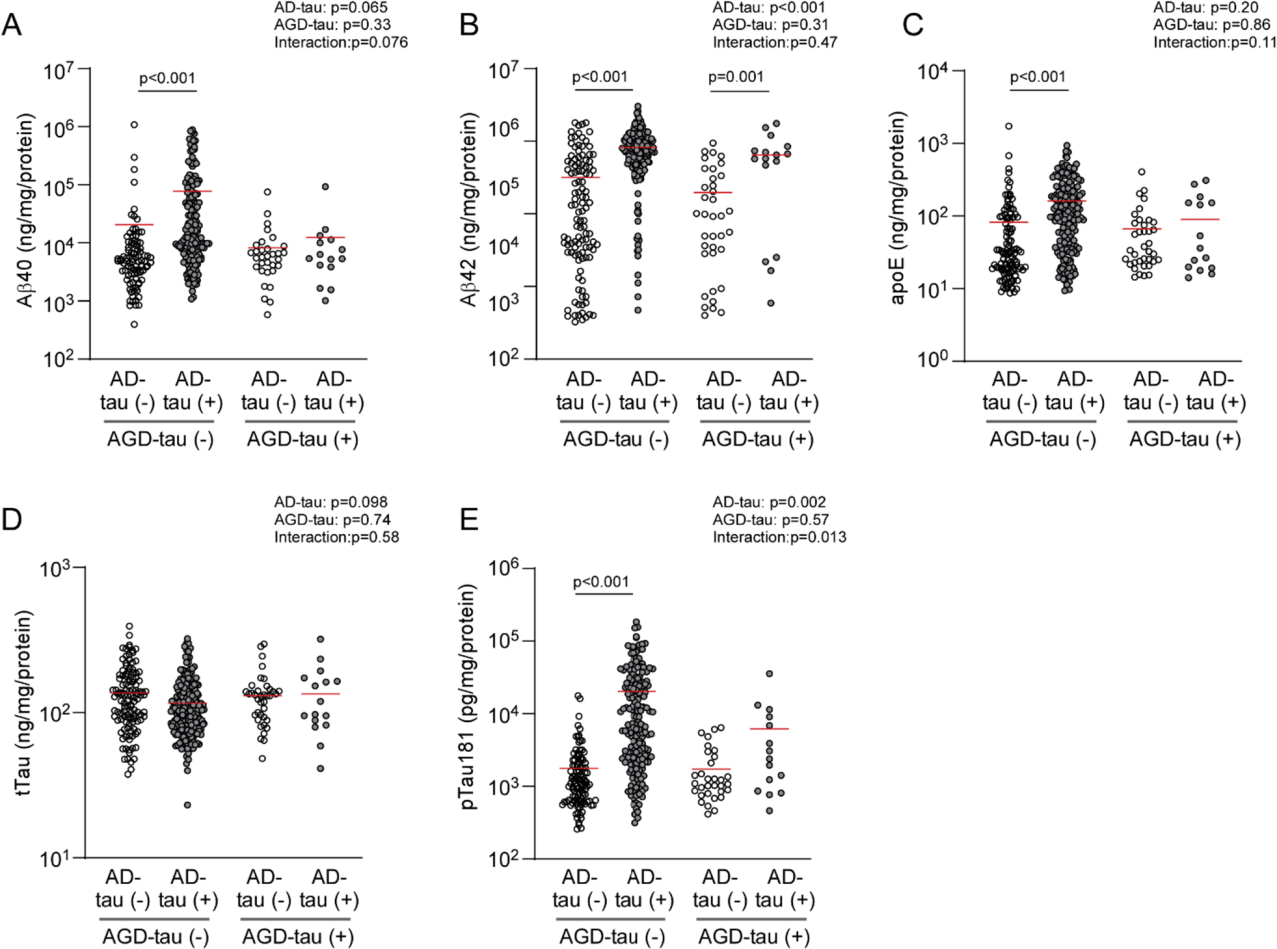
Insoluble AD-related molecule levels according to AD-tau and AGD-tau pathology. Dot plots and the median for insoluble Aβ40 (**A**), Aβ42 (**B**), apoE (**C**), tTau (**D**), and pTau181 (**E**) levels in FA fraction are shown according to AD-tau and AGD-tau pathology. Measures of AD-related molecules were normalized by corresponding protein concentrations in each sample. *P*-values result from linear regression models that were adjusted for age and sex

When segregating cases based on the presence or absence of AD-tau or AGD-tau pathology (Additional file 1: Table S7), cases with AD-tau pathology had higher levels of insoluble Aβ40, Aβ42, apoE, and pTau181 compared to those without AD-tau, particularly in the absence of AGD-tau (Fig. 2). Though not a significant interaction (P < 0.0056 considered as significant), it is worth noting that significant positive associations of AD-tau with insoluble Aβ40 (FA: β = 12.97, *p* < 0.001), insoluble apoE (FA: β = 1.05, *p* < 0.001), and insoluble pTau181 (FA: β = 1.06, *p* < 0.001) were observed in AGD-tau negative cases, but not in positive cases (Additional file 1: Table S8). AD-tau was also positively associated with insoluble Aβ42 levels, regardless of the AGD stratification (AGD-tau negative: β = 34.95, *p* < 0.001; AGD-tau positive: β = 28.34, *p* = 0.001). However, AD-tau association to insoluble Aβ42 levels is slightly weaker in the presence of AGD-tau pathology. Although not quite significant, the presence of AGD-tau was negatively associated with insoluble levels of Aβ40 (FA: β = − 9.96, *p* = 0.070), Aβ42 (FA: β = − 13.29, *p* = 0.046) and pTau181 (FA: β = − 0.68, *p* = 0.026) after adjusting for age and sex in AD-tau positive cases. There were no significant interactions between AD-tau and AGD-tau. Neuritic plaque score significantly interacted with AD-tau for insoluble pTau181 levels (Additional file 1: Table S9).

**Fig. 3:**
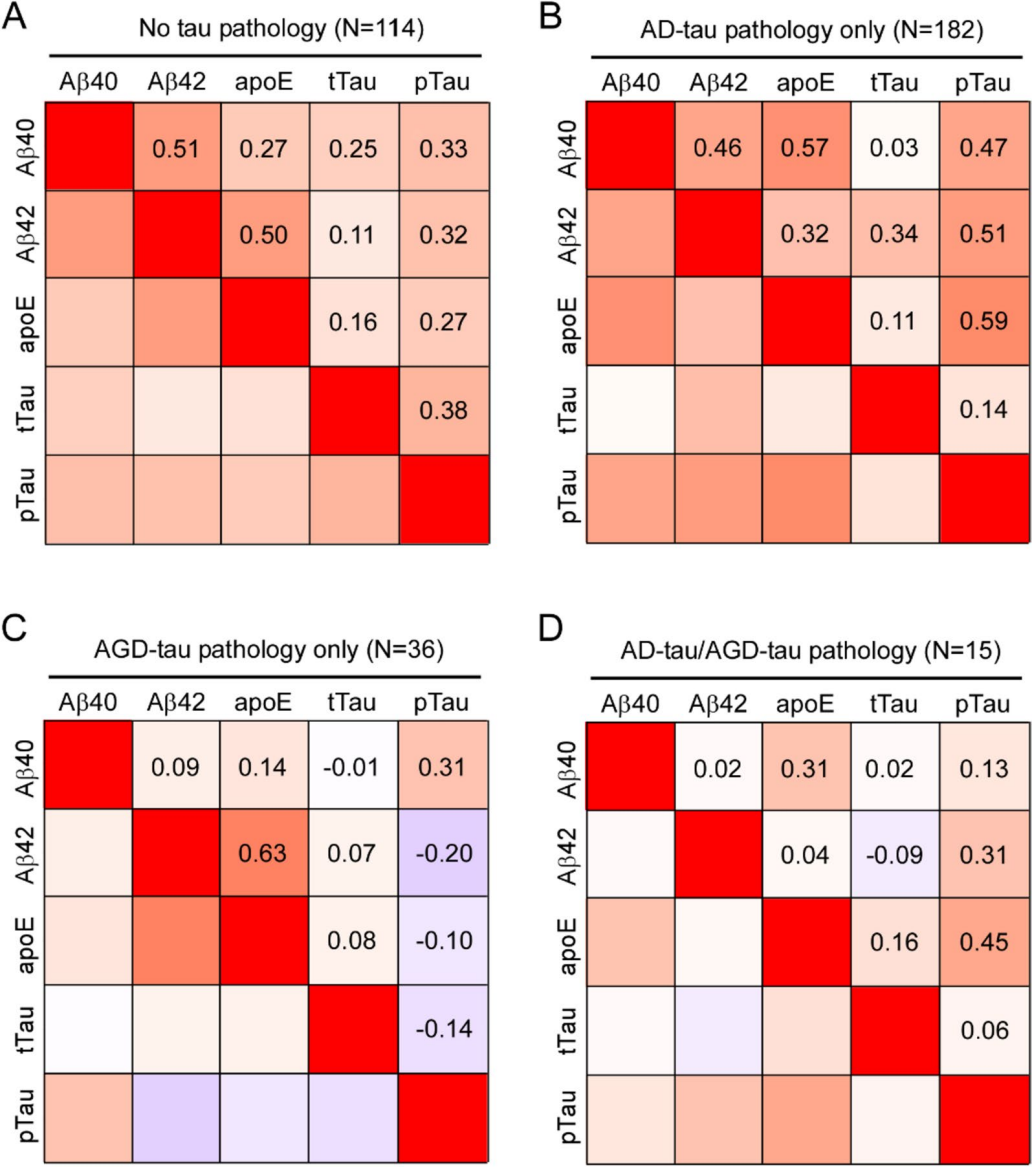
Correlation matrices of insoluble AD-related molecule levels according to AD-tau and AGD-tau pathology. Heatmap of Spearman correlation among insoluble Aβ40, Aβ42, apoE, tTau, and pTau181 levels in FA fraction are shown by stratifying to groups for (**A**) no tau pathology, (**B**) AD-tau pathology only, (**C**) AGD-tau pathology only, and (**D**) AD-tau and AGD-tau pathology. Correlation coefficients are visualized with blue-red gradients (− 1.0 to 1.0) and the numbers in the cells represent Spearman’s r

When investigating multivariate correlations among the insoluble AD-related molecules, we found differences in the strength and direction of the associations depending on tau pathology status. Levels of insoluble pTau181 were positively correlated with levels of Aβ40, Aβ42, and apoE in the AD-tau only pathology group. The strength of these associations was weaker in the no tau pathology group and in the AD-tau/AGD-tau group. While insoluble pTau181 and insoluble Aβ40 remained positively corelated in the AGD-tau only group, insoluble pTau181 levels were inversely correlated with levels of insoluble Aβ42 and apoE. Although a positive association was detected between the levels of insoluble tTau and the levels of insoluble Aβ42 in the AD-tau only group, this association was weaker in the no tau pathology group and in the AGD-tau only group, and it was reversed in the AD-tau/AGD-tau group. Overall, the strength of the associations between the insoluble AD-related molecules are modest in the AD-tau/AGD-tau group compared to the AD-tau only group (Fig. 3).

### *APOE4* is associated with AD-related molecules in the absence of AGD-tau

We then examined the effects of *APOE* genotype according to AD-tau/AGD-tau pathology on insoluble levels of AD-related molecules through linear regression analyses adjusted for age and sex (Table 5). In the group without AD-tau and AGD-tau pathology, *APOE4* was significantly associated with higher levels of soluble, detergent-soluble, and insoluble Aβ42 compared to *APOE3* (TBS: β = 2.68, *p* < 0.001; TBSX: β = 2.18, *p* = 0.002; FA: β = 21.79, *p* = 0.006). In the AD-tau positive group without AGD-tau pathology, higher levels of insoluble Aβ40 (FA: β = 14.15, *p* < 0.001), apoE (FA: β = 1.41, *p* < 0.001), and pTau181 (FA: β = 0.60, *p* = 0.001) as well as detergent-soluble Aβ40 (TBSX: β = 1.31, *p* = 0.002), Aβ42 (TBSX: β = 1.07, *p* = 0.004) and apoE (TBSX: β = 0.22, *p* = 0.002) were observed in the *APOE4* group compared to the *APOE3* group. Weaker associations between *APOE4* and the levels of insoluble Aβ40 and of pTau 181 were in the AGD-tau pathology positive group.

**Table 5:** Interactions of *APOE* with tau pathology regarding associations with AD-related molecules

	N	No tau pathology: AD-tau ( −), AGD-tau ( −)		AD-tau pathology only AD-tau ( +), AGD-tau ( −)		Mixed tauopathy AD-tau (+ / −), AGD-tau ( +)		*APOE* x tau pathology interaction
		β (95% CI)	*P*-value	β (95% CI)	*P*-value	β (95% CI)	P-value	Interaction *p*-value
*APOE2* vs. *APOE3* (reference)		*APOE2* (N = 21) vs *APOE3* (N = 73)		*APOE2* (N = 13) vs *APOE3* (N = 54)		*APOE2* (N = 11) vs *APOE3* (N = 27)		
Aβ40-TBS	174	0.90 (− 0.20 to 1.99)	0.11	0.12 (− 1.26 to 1.49)	0.86	− 0.72 (− 2.08–0.63)	0.28	0.45
Aβ40-TBSX	188	0.27 (− 0.51 to 1.05)	0.49	− 0.32 (− 1.41 to 0.77)	0.56	− 0.94 (− 1.80, 0.08)	0.034	0.14
Aβ40-FA	188	1.47 (− 3.72 to 6.67)	0.57	1.16 (− 7.28 to 9.61)	0.78	3.05 (− 2.25 to 8.34)	0.25	0.95
Aβ42-TBS	186	0.09 (− 1.38 to 1.56)	0.90	1.52 (− 0.73 to 3.76)	0.18	0.42 (− 1.86 to 2.70)	0.71	0.60
Aβ42-TBSX	192	− 0.19 (− 1.45 to 1.07)	0.77	0.00 (− 1.84 to 1.84)	1.00	− 2.47 (− 4.64, 0.30)	0.027	0.11
Aβ42-FA	197	1.86 (− 12.81 to 16.52)	0.80	6.19 (− 11.78 to 24.17)	0.49	− 3.16 (− 25.26 to 18.93)	0.77	0.77
apoE-TBS	196	0.52 (0.02 to 1.03)	0.042	0.56 (− 0.02 to 1.15)	0.059	0.75 (0.18to 1.32)	0.012	0.90
apoE-TBSX	194	− 0.04 (− 0.27 to 0.18)	0.70	− 0.17 (− 0.43 to 0.08)	0.18	− 0.19 (− 0.52 to 0.14)	0.26	0.64
apoE-FA	197	0.17 (− 0.59 to 0.93)	0.66	0.06 (− 0.88 to 1.00)	0.90	0.07 (− 0.81 to 0.95)	0.87	0.89
tTau-TBS	176	− 7.27 (-14.55, 0.00)	0.050	3.50 (-6.45 to 13.44)	0.48	− 1.75 (− 12.13 to 8.64)	0.73	0.18
tTau-TBSX	194	− 0.05 (− 0.25 to 0.16)	0.67	0.08 (− 0.24 to 0.39)	0.63	− 0.19 (− 0.46 to 0.09)	0.18	0.51
tTau-FA	201	− 0.05 (− 0.30 to 0.20)	0.69	**0.35 (0.10 to 0.60)**	**0.007**	0.25 (− 0.06 to 0.55)	0.11	0.061
pTau181-TBS	194	− 0.01 (− 0.25 to 0.22)	0.92	− 0.08 (− 0.41 to 0.24)	0.61	− 0.34 (− 0.72 to 0.04)	0.075	0.23
pTau181-TBSX	194	− 0.19 (− 0.38 to 0.01)	0.058	0.10 (− 0.10 to 0.31)	0.32	− 0.20 (− 0.46 to 0.06)	0.13	0.10
pTau181-FA	192	− 0.24 (− 0.44, 0.03)	0.023	0.46 (− 0.18 to 1.10)	0.15	0.24 (− 0.14 to 0.62)	0.21	0.065
*APOE4* vs. *APOE3* (reference)		*APOE4* (N = 20) vs *APOE3* (N = 73)		*APOE4* (N = 115) vs *APOE3* (N = 54)		*APOE4* (N = 13) vs *APOE3* (N = 27)		
Aβ40-TBS	267	0.48 (− 0.52 to 1.49)	0.34	0.85 (− 0.07 to 1.77)	0.069	− 0.63 (− 1.98 to 0.71)	0.34	0.50
Aβ40-TBSX	292	0.12 (− 0.68 to 0.93)	0.76	**1.31 (0.51 to 2.11)**	**0.002**	− 0.14 (− 0.74 to 0.47)	0.65	0.049
Aβ40-FA	290	7.21 (− 0.05 to 14.48)	0.052	**14.15 (7.47 to 20.83)**	** < 0.001**	1.44 (− 3.49 to 6.37)	0.56	0.088
Aβ42-TBS	289	**2.68 (1.34 to 4.02)**	** < 0.001**	1.08 (0.19 to 1.96)	0.017	2.09 (− 0.13 to 4.32)	0.064	0.11
Aβ42-TBSX	293	**2.18 (0.80 to 3.57)**	**0.002**	**1.07 (0.36 to 1.79)**	**0.004**	**2.98 (0.99 to 4.97)**	**0.004**	0.10
Aβ42-FA	303	**21.79 (6.52 to 37.07)**	**0.006**	9.15 (1.22 to 17.07)	0.024	**26.52 (7.49 to 45.55)**	**0.008**	0.16
apoE-TBS	303	− 0.29 (− 0.80 to 0.22)	0.26	− 0.33 (− 0.65, 0.02)	0.036	0.27 (− 0.25 to 0.79)	0.30	0.32
apoE-TBSX	296	− 0.03 (− 0.28 to 0.22)	0.82	**0.22 (0.08 to 0.36)**	**0.002**	0.02 (− 0.27 to 0.31)	0.89	0.087
apoE-FA	302	0.31 (− 0.46 to 1.08)	0.43	**1.41 (0.82 to 2.01)**	** < 0.001**	0.91 (− 0.15 to 1.96)	0.091	0.085
tTau-TBS	278	− 0.34 (− 7.37 to 6.69)	0.92	1.01 (− 4.32 to 6.34)	0.71	− 1.23 (− 10.56 to 8.09)	0.79	0.85
tTau-TBSX	296	0.01 (− 0.21 to 0.23)	0.91	− 0.09 (− 0.26 to 0.07)	0.25	0.05 (− 0.23 to 0.33)	0.71	0.76
tTau-FA	308	0.02 (− 0.23 to 0.27)	0.89	− 0.09 (− 0.23 to 0.05)	0.19	− 0.08 (− 0.38 to 0.22)	0.60	0.76
pTau181-TBS	297	− 0.11 (− 0.36 to 0.13)	0.37	− 0.03 (− 0.20 to 0.15)	0.77	− 0.18 (− 0.42 to 0.07)	0.16	0.79
pTau181-TBSX	295	− 0.23 (− 0.44, 0.03)	0.024	0.09 (− 0.02 to 0.21)	0.10	− 0.22 (− 0.44 to 0.00)	0.052	**0.004**
pTau181-FA	296	0.18 (− 0.04–0.40)	0.12	**0.60 (0.24 to 0.97)**	**0.001**	0.04 (− 0.33 to 0.40)	0.85	0.067

*β* Regression coefficient; *CI* Confidence interval. β values, 95% CIs, and p-values result from linear regression models that were adjusted for age and sex. β values are interpreted as the difference in means of the given AD-related molecule on the square root (tTau-TBS), cube root (apoE-TBSX, tTau-TBSX, tTau-FA), or natural logarithm scale (Aβ40-TBS, Aβ40-TBSX, Aβ40-FA, Aβ42-TBS, Aβ42-TBSX, Aβ42-FA, apoE-TBS, apoE-FA, pTau181-TBS, pTau181-TBSX, pTau181-FA) in comparison to the *APOE3* group. For tests of interaction, models were additionally adjusted for *APOE* group and the interaction between combination of AD-tau and AGD-tau pathology and *APOE* group. *P*-values < 0.01 were considered as statistically significant after applying a Bonferroni correction for multiple testing separately for each fraction and each pair-wise comparison between *APOE* groups; significant associations are underlined, and significant interactions are shown in bold

*APOE4* remained significantly associated with detergent-soluble and insoluble Aβ42 levels compared to *APOE3* in the mixed tau pathology group (TBSX: β = 2.98, *p* = 0.004; FA: β = 26.52, *p* = 0.008), which may be driven by AD-tau positivity as, out of 13 cases, 12 are AD-tau positive (Table 5).

It is however important to note that no significant interactive effects were reached between *APOE4* and tau pathology, aside from a significant interaction between levels of detergent-soluble pTau-181 and *APOE*4. Further, there were no significant differences in levels of AD-related molecules between *APOE2* and *APOE3* groups irrespective of tau pathology stratification (Table 5).

## Discussion

Carrying *APOE4* significantly increases the risk of AD and age-related cognitive decline [30, 40]. While *APOE* genotype appears to influence AD pathogenesis through multiple pathways, the predominant effect in modulating amyloid pathology has been implicated as a major mechanism impacting AD risk [19]. A meta-analysis in non-dementia cohorts has shown that amyloid positivity, determined through amyloid PET imaging and CSF biomarkers, is exacerbated during aging in an *APOE* genotype-dependent manner (ε4/ε4 > ε3/ε4 = ε2/ε4 > ε3/ε3 > ε2/ε3 > ε2/ε2) [16]. In addition to Aβ [31], *APOE4* has been implicated to influence proteinopathies involving tau, α-synuclein, and TDP-43 [7, 9]. Indeed, in this study we also found that *APOE4* is associated with prevalence of AD-tau pathology as well as worsen amyloid score in our cohort composed of cognitively unimpaired individuals, individuals with mild cognitive impairment, and AD cases. Moreover, major AD-related molecules including insoluble Aβ40, Aβ42, apoE, and pTau181 were significantly increased in the presence of *APOE4*. However, associations of Aβ40 and pTau181 with *APOE4* were no longer evident in the presence of AGD-tau. Consistent with previous studies [36, 38], our cohort had a lower percentage of *APOE4* carriers in cases with AGD-tau pathology. Although AGD is a common tauopathy frequently detected in AD [37, 41], there is likely a distinct role of *APOE4* in tau pathogenesis between AD-tau and AGD-tau. On the one hand, *APOE4* may facilitate the shift from AGD-tau to AD-tau, while it is also possible that AGD-tau somehow mitigates the deleterious *APOE4* effects exacerbating AD-related pathology. On the other hand, there was a trend increase of AGD-tau pathology in the presence of *APOE2* which is consistent with a previous report [8]. Interestingly, polymorphisms in α2-macroglobulin (*A2M*) and low-density lipoprotein receptor-related protein 1 (*LRP1*) genes are also associated with AGD risk [10]. While LRP1 functions as a receptor for apoE and α2M, it has also been shown to mediate the cellular uptake and propagation of tau [25]. Thus, the apoE-LRP1 axis may be involved in the molecular mechanism mediating the development of AD-tau or AGD-tau pathology.

Synergistic effects between Aβ and tau in AD pathogenesis have been compellingly recognized [4]. We also found positive associations among insoluble Aβ40, Aβ42, apoE, and pTau181 levels in AD-tau positive cases without AGD-tau. However, these associations were either weakened in co-occurring AGD-tau and AD-tau cases, or even reversed to negative associations in the presence of only AGD-tau. Since the tauopathy negative group (without both AD-tau or AGD-tau) also showed associations among Aβ, apoE, and pTau181, their interactions are likely diminished through unknown mechanisms in AGD-tau positive cases. Weaker associations between Aβ40, Aβ42, and apoE are observed in the presence of AGD-tau, possibly indicating that AGD might cause tauopathy independently of Aβ. The balance of Aβ-apoE-tau interaction may be a key factor influencing the development of either AD-tau or AGD-tau pathology, or their co-occurrence. The conflicting *APOE4* effects on AD-tau and AGD-tau pathologies may be due to its proneness facilitating the proteinopathy in the brain. The structure of 4R-tau filaments in AGD differs from those from AD [32]. The tau properties of AGD-tau may induce the suppressive effects on Aβ and apoE aggregation although further studies are needed. In addition, Aβ has been shown to accelerate tau propagation from the entorhinal cortex and medial temporal lobe into limbic system and neocortex through the hippocampal cingulum bundle [4, 14, 15]. In most AGD cases, tauopathy is detected in ambient gyrus, hippocampus, anterior entorhinal area and amygdala (Stage I), but spreads into medial temporal lobe and subiculum (Stage II), and to anterior temporal, cingulate gyrus, rectus gyrus, septum, accumbens nucleus, insular and orbitofrontal cortices, and hypothalamus (Stage III) [29]. Since the AGD stages are not associated with Braak stages and Thal phase [29], AGD tauopathy is predicted to propagate through an Aβ-independent manner. Co-occurrence of AGD and AD may affect the nature of tau properties and consequently its spread. Of note, a recent study has identified *APOE* as one of the top-ranked genes whose expression is associated with the spatial spreading of tau [20]. Thus, apoE amounts as well as *APOE* genotype may also differently influence the development of AD-tau and AGD-tau pathologies. In addition, co-occurring limbic predominant age-related TDP-43 encephalopathy neuropathological changes (LATE-NC) in AD has been suggested to associate with elevated tau levels [39]. However, our biochemical analyses in the medial temporal cortex did not reveal significant correlations between tau levels (tTau or pTau181) and neuropathologically defined TDP-43 pathology. This discrepancy may be due to our measurements differing both in brain region (medial temporal cortex as opposed to entorhinal and frontal cortex) and phosphorylated tau isoform (p-Tau 181 as opposed to p-Tau 199). Moreover, the lack of TDP-43 biochemical measures in our study emphasizes the need for even more comprehensive investigations across varied brain regions and tau isoforms to further explore the relationship between LATE-NC and AD.

Our study showed that the presence of AD-tau or AGD-tau pathologies differentially influences the cognitive functions assessed by MMSE. The occurrence of AD-tau pathology was negatively associated with MMSE scores. However, the significant association between AD-tau and MMSE score was weakened in the presence of AGD-tau. This result is in line with another study reporting that cognitive status is not affected by the presence of AGD [13]. Since Aβ and tau synergically cause synaptic damage and neurodegeneration [4], lower Aβ accumulation and lack of Aβ-tau interaction in AGD-tauopathy may be involved in the benign effects on cognitive function even in the presence of AD-tau. In addition, tau acetylation at K274 residue was not detected in AGD-tau, while this specific posttranslational modification was generally identified in other tauopathies [11]. Since tau K274 acetylation exacerbates tau aggregation and cytotoxicity [24], the unique nature of AGD-tau may mitigate AD-tau toxicity. However, cognitive function is likely impaired in severe AGD cases. At AGD stage III, 71.2% of cases have been reported to have dementia with the Clinical Dementia Rating (CDR) ≥ 1 [29]. Since Braak stages and Thal phase are milder in dementia cases with AGD compared to AD [35], the mechanisms of neuronal damages caused by AGD-tau should differ from those of AD-tau. While AGD-tau may be preventive against AD-related phenotypes by lowering tau aggregation and propagation at Stage I, the wide-spread AGD-tau at Stage III may cause cognitive decline independently of the more common amyloid and tau pathology detected in AD. It is worth noting that although the lack of a relationship found between the presence of AGD and cognitive impairment agrees with past literature, we did not specifically apply Saito staging to evaluate regional involvement of argyrophilic grains [13, 22, 27, 28].

In summary, we demonstrated that *APOE4* increases the risk of AD-tau pathology, but not AGD-tau pathology, accompanied with exacerbated accumulation of insoluble Aβ40, Aβ42, apoE and pTau181. In the presence of AGD-tau, the effect of AD-tau on cognitive impairment became modest with lower insoluble AD-related molecule levels and a lack of association amongst those molecules. Our study provides a comprehensive analysis into how *APOE* genotype influences the trajectory of AD-tau and AGD-tau pathologies by incorporating biochemical measures, thus supplementing, and enriching our understanding of the neuropathological studies previously published. However, with our study predominantly presenting association data, experimental validation in future work will strengthen the robustness of our findings. One limitation of our study is that we subjectively built the cohort based on *APOE* genotype. Since *APOE2* and *APOE4* carriers in our cohort are more prominent than in the general population, their effects may be over- or under-estimated in our study. There is also a possibility of a false-negative error due to the relatively small sample numbers. Further studies should define interactions among *APOE*, AGD-tau and AD-tau by including various brain regions and assessing other phosphorylated tau species, ideally in a larger cohort with different stages of AGD, spanning different ages and *APOE* genotypes.

### Supplementary Information

**Additional file 1.** Table S1: Associations of neuropathological measures with MMSE score. Table S2: Assessment of interactions of AGD-tau or AD-tau with neuropathological measures regarding associations with MMSE score. Table S3: Descriptive summaries of AD-related molecules levels according to *APOE* genotype. Table S4: Associations of MMSE score with AD-related molecule levels. Table S5: Associations of neuropathological measures with AD-related molecules (TBS). Table S6: Associations of neuropathological measures with AD-related molecules (TBSX). Table S7: Subject characteristics according to combination of AD-tau and AGD-tau pathology. Table S8: Interactions of AGD-tau with neuropathological measures regarding associations with AD-related molecules. Table S9: Interactions of AD-tau with neuropathological measures regarding associations with AD-related molecules.

## References

[1] Braak H, Alafuzoff I, Arzberger T, Kretzschmar H, Del Tredici K (2006). Staging of Alzheimer disease-associated neurofibrillary pathology using paraffin sections and immunocytochemistry. Acta Neuropathol.

[2] Braak H, Braak E (1991). Neuropathological stageing of Alzheimer-related changes. Acta Neuropathol.

[3] Brenowitz WD, Hubbard RA, Keene CD, Hawes SE, Longstreth WT, Woltjer RL, Kukull WA (2017). Mixed neuropathologies and estimated rates of clinical progression in a large autopsy sample. Alzheimers Dement.

[4] Busche MA, Hyman BT (2020). Synergy between amyloid-beta and tau in Alzheimer's disease. Nat Neurosci.

[5] Das P, Verbeeck C, Minter L, Chakrabarty P, Felsenstein K, Kukar T, Maharvi G, Fauq A, Osborne BA, Golde TE (2012). Transient pharmacologic lowering of Abeta production prior to deposition results in sustained reduction of amyloid plaque pathology. Mol Neurodegener.

[6] DeTure MA, Dickson DW (2019). The neuropathological diagnosis of Alzheimer's disease. Mol Neurodegener.

[7] Frisoni GB, Altomare D, Thal DR, Ribaldi F, van der Kant R, Ossenkoppele R, Blennow K, Cummings J, van Duijn C, Nilsson PM (2022). The probabilistic model of Alzheimer disease: the amyloid hypothesis revised. Nat Rev Neurosci.

[8] Ghebremedhin E, Schultz C, Botez G, Rub U, Sassin I, Braak E, Braak H (1998). Argyrophilic grain disease is associated with apolipoprotein E epsilon 2 allele. Acta Neuropathol.

[9] Ghebremedhin E, Schultz C, Braak E, Braak H (1998). High frequency of apolipoprotein E epsilon4 allele in young individuals with very mild Alzheimer's disease-related neurofibrillary changes. Exp Neurol.

[10] Ghebremedhin E, Schultz C, Thal DR, Del Tredici K, Rueb U, Braak H (2002). Genetic association of argyrophilic grain disease with polymorphisms in alpha-2 macroglobulin and low-density lipoprotein receptor-related protein genes. Neuropathol Appl Neurobiol.

[11] Grinberg LT, Wang X, Wang C, Sohn PD, Theofilas P, Sidhu M, Arevalo JB, Heinsen H, Huang EJ, Rosen H (2013). Argyrophilic grain disease differs from other tauopathies by lacking tau acetylation. Acta Neuropathol.

[12] Hixson JE, Vernier DT (1990). Restriction isotyping of human apolipoprotein E by gene amplification and cleavage with HhaI. J Lipid Res.

[13] Iida MA, Farrell K, Walker JM, Richardson TE, Marx GA, Bryce CH, Purohit D, Ayalon G, Beach TG, Bigio EH (2021). Predictors of cognitive impairment in primary age-related tauopathy: an autopsy study. Acta Neuropathol Commun.

[14] Jacobs HIL, Hedden T, Schultz AP, Sepulcre J, Perea RD, Amariglio RE, Papp KV, Rentz DM, Sperling RA, Johnson KA (2018). Structural tract alterations predict downstream tau accumulation in amyloid-positive older individuals. Nat Neurosci.

[15] Jagust W (2018). Imaging the evolution and pathophysiology of Alzheimer disease. Nat Rev Neurosci.

[16] Jansen WJ, Ossenkoppele R, Knol DL, Tijms BM, Scheltens P, Verhey FRJ, Visser PJ, Group at ABS (2015). Prevalence of cerebral amyloid pathology in persons without dementia: a meta-analysis. JAMA.

[17] Li Z, Shue F, Zhao N, Shinohara M, Bu G (2020). APOE2: protective mechanism and therapeutic implications for Alzheimer's disease. Mol Neurodegener.

[18] Liu CC, Yamazaki Y, Heckman MG, Martens YA, Jia L, Yamazaki A, Diehl NN, Zhao J, Zhao N, DeTure M (2020). Tau and apolipoprotein E modulate cerebrovascular tight junction integrity independent of cerebral amyloid angiopathy in Alzheimer's disease. Alzheimers Dement.

[19] Martens YA, Zhao N, Liu CC, Kanekiyo T, Yang AJ, Goate AM, Holtzman DM, Bu G (2022). ApoE Cascade Hypothesis in the pathogenesis of Alzheimer's disease and related dementias. Neuron.

[20] Montal V, Diez I, Kim CM, Orwig W, Bueicheku E, Gutierrez-Zuniga R, Bejanin A, Pegueroles J, Dols-Icardo O, Vannini P (2022). Network Tau spreading is vulnerable to the expression gradients of APOE and glutamatergic-related genes. Sci Transl Med.

[21] Montine TJ, Phelps CH, Beach TG, Bigio EH, Cairns NJ, Dickson DW, Duyckaerts C, Frosch MP, Masliah E, Mirra SS (2012). National Institute on Aging-Alzheimer's Association guidelines for the neuropathologic assessment of Alzheimer's disease: a practical approach. Acta Neuropathol.

[22] Nelson PT, Abner EL, Schmitt FA, Kryscio RJ, Jicha GA, Smith CD, Davis DG, Poduska JW, Patel E, Mendiondo MS (2010). Modeling the association between 43 different clinical and pathological variables and the severity of cognitive impairment in a large autopsy cohort of elderly persons. Brain Pathol.

[23] Nguyen AT, Kouri N, Labuzan SA, Przybelski SA, Lesnick TG, Raghavan S, Reid RI, Reichard RR, Knopman DS, Petersen RC (2022). Neuropathologic scales of cerebrovascular disease associated with diffusion changes on MRI. Acta Neuropathol.

[24] Rane JS, Kumari A, Panda D (2019). An acetylation mimicking mutation, K274Q, in tau imparts neurotoxicity by enhancing tau aggregation and inhibiting tubulin polymerization. Biochem J.

[25] Rauch JN, Luna G, Guzman E, Audouard M, Challis C, Sibih YE, Leshuk C, Hernandez I, Wegmann S, Hyman BT (2020). LRP1 is a master regulator of tau uptake and spread. Nature.

[26] Raulin AC, Doss SV, Trottier ZA, Ikezu TC, Bu G, Liu CC (2022). ApoE in Alzheimer's disease: pathophysiology and therapeutic strategies. Mol Neurodegener.

[27] Rodriguez RD, Suemoto CK, Molina M, Nascimento CF, Leite RE, de Lucena Ferretti-Rebustini RE, Farfel JM, Heinsen H, Nitrini R, Ueda K (2016). Argyrophilic Grain disease: demographics, clinical, and neuropathological features from a large autopsy study. J Neuropathol Exp Neurol.

[28] Sabbagh MN, Agro A, Bell J, Aisen PS, Schweizer E, Galasko D (2011). PF-04494700, an oral inhibitor of receptor for advanced glycation end products (RAGE), in Alzheimer disease. Alzheimer Dis Assoc Disord.

[29] Saito Y, Ruberu NN, Sawabe M, Arai T, Tanaka N, Kakuta Y, Yamanouchi H, Murayama S (2004). Staging of argyrophilic grains: an age-associated tauopathy. J Neuropathol Exp Neurol.

[30] Saunders AM, Strittmatter WJ, Schmechel D, George-Hyslop PH, Pericak-Vance MA, Joo SH, Rosi BL, Gusella JF, Crapper-MacLachlan DR, Alberts MJ (1993). Association of apolipoprotein E allele epsilon 4 with late-onset familial and sporadic Alzheimer's disease. Neurology.

[31] Schmechel DE, Saunders AM, Strittmatter WJ, Crain BJ, Hulette CM, Joo SH, Pericak-Vance MA, Goldgaber D, Roses AD (1993). Increased amyloid beta-peptide deposition in cerebral cortex as a consequence of apolipoprotein E genotype in late-onset Alzheimer disease. Proc Natl Acad Sci U S A.

[32] Shi Y, Zhang W, Yang Y, Murzin AG, Falcon B, Kotecha A, van Beers M, Tarutani A, Kametani F, Garringer HJ (2021). Structure-based classification of tauopathies. Nature.

[33] Shinohara M, Murray ME, Frank RD, Shinohara M, DeTure M, Yamazaki Y, Tachibana M, Atagi Y, Davis MD, Liu CC (2016). Impact of sex and APOE4 on cerebral amyloid angiopathy in Alzheimer's disease. Acta Neuropathol.

[34] Thal DR, Rub U, Orantes M, Braak H (2002). Phases of A beta-deposition in the human brain and its relevance for the development of AD. Neurology.

[35] Thal DR, Schultz C, Botez G, Del Tredici K, Mrak RE, Griffin WS, Wiestler OD, Braak H, Ghebremedhin E (2005). The impact of argyrophilic grain disease on the development of dementia and its relationship to concurrent Alzheimer's disease-related pathology. Neuropathol Appl Neurobiol.

[36] Togo T, Cookson N, Dickson DW (2002). Argyrophilic grain disease: neuropathology, frequency in a dementia brain bank and lack of relationship with apolipoprotein E. Brain Pathol.

[37] Togo T, Sahara N, Yen SH, Cookson N, Ishizawa T, Hutton M, de Silva R, Lees A, Dickson DW (2002). Argyrophilic grain disease is a sporadic 4-repeat tauopathy. J Neuropathol Exp Neurol.

[38] Tolnay M, Probst A, Monsch AU, Staehelin HB, Egensperger R (1998). Apolipoprotein E allele frequencies in argyrophilic grain disease. Acta Neuropathol.

[39] Tome SO, Tsaka G, Ronisz A, Ospitalieri S, Gawor K, Gomes LA, Otto M, von Arnim CAF, Van Damme P, Van Den Bosch L (2023). TDP-43 pathology is associated with increased tau burdens and seeding. Mol Neurodegener.

[40] Yamazaki Y, Zhao N, Caulfield TR, Liu CC, Bu G (2019). Apolipoprotein E and Alzheimer disease: pathobiology and targeting strategies. Nat Rev Neurol.

[41] Zhang Y, Wu KM, Yang L, Dong Q, Yu JT (2022). Tauopathies: new perspectives and challenges. Mol Neurodegener.

