# Supplementary material for: Impact of APOE on amyloid and tau accumulation in argyrophilic grain disease and Alzheimer’s disease
Source: Acta Neuropathol Commun. 2024 Feb 9;12:25. doi: 10.1186/s40478-024-01731-0 (PMC10854035; doi:10.1186/s40478-024-01731-0)
Supplement: Supplementary file 1 — Additional file 1. Table S1: Associations of neuropathological measures with MMSE score. Table S2: Assessment of interactions of AGD-tau or AD-tau with neuropathological measures regarding associations with MMSE score. Table S3: Descriptive summaries of AD-related molecules levels according to APOE genotype. Table S4: Associations of MMSE score with AD-related molecule levels. Table S5: Associations of neuropathological measures with AD-related molecules (TBS). Table S6: Associations of neuropathological measures with AD-related molecules (TBSX). Table S7: Subject characteristics according to combination of AD-tau and AGD-tau pathology. Table S8: Interactions of AGD-tau with neuropathological measures regarding associations with AD-related molecules. Table S9: Interactions of AD-tau with neuropathological measures regarding associations with AD-related molecules. [file 40478_2024_1731_MOESM1_ESM.docx]

**Table S1: Associations of neuropathological measures with MMSE score**

|  |  | Unadjusted analysis | | Adjusting for age and sex | | Full multivariable analysis | |
| --- | --- | --- | --- | --- | --- | --- | --- |
| Variable | N | β (95% CI) | P-value | β (95% CI) | P-value | β (95% CI) | P-value |
| Age | 133 | **-1.34 (-2.08, -0.59)** | **<0.001** | **-1.33 (-2.08, -0.59)** | **<0.001** | **-1.07 (-1.79, -0.35)** | **0.004** |
| Sex | 133 | 0.19 (-0.79, 1.18) | 0.70 | 0.11 (-0.83, 1.06) | 0.81 | 0.10 (-0.79, 1.00) | 0.82 |
| *APOE4* | 133 | -0.71 (-1.86, 0.44) | 0.22 | -0.61 (-1.72, 0.50) | 0.28 | 0.01 (-1.10, 1.11) | 1.00 |
| VaD | 133 | -0.62 (-1.63, 0.38) | 0.22 | -0.51 (-1.50, 0.47) | 0.30 | -0.24 (-1.18, 0.70) | 0.62 |
| CAA | 133 | -1.15 (-2.84, 0.55) | 0.18 | -1.04 (-2.68, 0.59) | 0.21 | -1.08 (-2.69, 0.54) | 0.19 |
| Neuritic plaque score | 133 | -0.64 (-1.13, -0.16) | 0.010 | -0.48 (-0.96, 0.00) | 0.052 | -0.09 (-0.59, 0.41) | 0.72 |
| TDP-43 | 133 | -1.88 (-4.21, 0.45) | 0.11 | -1.73 (-4.02, 0.55) | 0.14 | -1.00 (-3.18, 1.19) | 0.37 |
| Synuclein | 133 | -1.00 (-2.28, 0.29) | 0.13 | -0.78 (-2.04, 0.48) | 0.22 | -1.28 (-2.48, -0.08) | 0.037 |
| AD-tau | 133 | **-2.33 (-3.29, -1.37)** | **<0.001** | -**2.11 (-3.05, -1.17)** | **<0.001** | **-2.03 (-3.06, -1.00)** | **<0.001** |
| AGD-tau | 133 | 0.16 (-1.05, 1.38) | 0.79 | 0.42 (-0.76, 1.60) | 0.48 | 0.27 (-0.86, 1.39) | 0.64 |
| β=regression coefficient; CI=confidence interval. β values, 95% CIs, and p-values result from linear regression models. β values are interpreted as the change in mean MMSE score corresponding to each 10-year increase in age, male sex, presence of *APOE* ε4, presence of VaD, presence of CAA, 1 unit increase in neuritic plaque score, presence of TDP-43 pathology, presence of synucleinopathy, presence of AD-tau or presence of AGD-tau. Full multivariable models were adjusted for age, sex, and all other variables with a p-value <0.05 in the unadjusted analysis (neuritic plaque score and AD tau). P-values <0.005 were considered as statistically significant after applying a Bonferroni correction for multiple testing; significant findings are shown in bold. | | | | | | | |

**Table S2: Assessment of interactions of AGD-tau or AD-tau with neuropathological measures regarding associations with MMSE score**

|  | No AGD-tau pathology (N=302) | | AGD tau-pathology (N=51) | |  |
| --- | --- | --- | --- | --- | --- |
| Variable | β (95% CI) | P-value | β (95% CI) | P-value | Interaction p-value |
| Association with MMSE score |  |  |  |  |  |
| Age | **-1.47 (-2.30, -0.64)** | **<0.001** | -0.39 (-2.33, 1.56) | 0.69 | 0.40 |
| Sex | 0.32 (-0.79, 1.44) | 0.56 | -0.70 (-2.66, 1.27) | 0.47 | 0.47 |
| *APOE4* | -0.83 (-2.09, 0.43) | 0.19 | 0.82 (-1.72, 3.35) | 0.51 | 0.32 |
| VaD | -0.87 (-1.99, 0.25) | 0.13 | 1.70 (-0.75, 4.15) | 0.16 | 0.064 |
| CAA | -1.10 (-3.05, 0.86) | 0.27 | -1.46 (-4.58, 1.66) | 0.34 | 1.00 |
| Neuritic plaque score | -0.47 (-1.01, 0.08) | 0.095 | -0.49 (-1.67, 0.69) | 0.40 | 0.80 |
| TDP-43 | -3.59 (-7.53, 0.35) | 0.074 | -1.80 (-4.70, 1.10) | 0.21 | 0.30 |
| Synuclein | -0.64 (-2.09, 0.82) | 0.39 | -1.41 (-4.12, 1.29) | 0.29 | 0.63 |
| AD-tau | **-2.56 (-3.61, -1.50)** | **<0.001** | -0.24 (-2.41, 1.92) | 0.82 | 0.042 |
|  | No AD tau-pathology (N=150) | | AD tau-pathology (N=197) | |  |
| Variable | β (95% CI) | P-value | β (95% CI) | P-value | Interaction p-value |
| Association with MMSE score |  |  |  |  |  |
| Age | -0.81 (-1.48, 0.14) | 0.018 | -1.75 (-3.62-0.12) | 0.066 | 0.21 |
| Sex | -0.13 (-1.01-0.74) | 0.76 | 0.42 (-1.73-2.56) | 0.70 | 0.45 |
| *APOE4* | -0.36 (-1.56-0.83) | 0.55 | 0.13 (-2.03-2.30) | 0.90 | 0.61 |
| VaD | 0.65 (-0.27-1.58) | 0.16 | -1.80 (-3.88-0.27) | 0.087 | 0.015 |
| CAA | -0.97 (-2.48-0.54) | 0.20 | -1.46 (-5.08-2.17) | 0.42 | 0.81 |
| Neuritic plaque score | -0.14 (-0.64-0.35) | 0.57 | 0.04 (-1.14-1.22) | 0.95 | 0.97 |
| TDP-43 | -0.37 (-3.34-2.61) | 0.81 | -1.40 (-5.09-2.29) | 0.45 | 0.58 |
| Synuclein | -0.85 (-1.92-0.22) | 0.12 | -2.98 (-6.61-0.66) | 0.11 | 0.14 |
| AGD-tau | -0.58 (-1.66-0.49) | 0.28 | 2.14 (-0.52-4.81) | 0.11 | 0.042 |
| β=regression coefficient; CI=confidence interval. β values, 95% CIs, and p-values result from linear regression models that were adjusted for age and sex. β values are interpreted as the change in mean MMSE score corresponding to each 10-year increase in age, male sex, presence of *APOE* ε4, presence of VaD, presence of CAA, 1 unit increase in neuritic plaque score, presence of TDP-43 pathology, presence of synucleinopathy, presence of AD-tau or presence of AGD-tau. P-values <0.0056 were considered as statistically significant after applying a Bonferroni correction for multiple testing separately for each AD-related molecule; significant associations are underlined, and significant interactions are shown in bold. | | | | | |

**Table S3: Descriptive summaries of AD-related molecules levels according to *APOE* genotype**

|  | *APOE2* (N=45) | | | *APOE3* (N=156) | | *APOE4* (N=152) | |
| --- | --- | --- | --- | --- | --- | --- | --- |
| Variable | N | Median (minimum, maximum) or No. (%) of patients | N | | Median (minimum, maximum) or No. (%) of patients | N | Median (minimum, maximum) or No. (%) of patients |
|  |  |  |  | |  |  |  |
| Aβ40 (pg/mg protein) |  |  |  | |  |  |  |
| TBS | 39 | 0.0 (0.0, 547.6) | 135 | | 0.0 (0.0, 250.2) | 132 | 14.0 (0.0, 3620.0) |
| TBSX | 40 | 66.6 (0.0, 822.1) | 148 | | 93.3 (0.0, 771.0) | 144 | 131.1 (0.0, 12823.1) |
| FA | 43 | 4095.2 (0.0, 141428.6) | 145 | | 5000.0 (0.0, 299062.5) | 145 | 11400.0 (0.0, 1087903.2) |
| Aβ42 (pg/mg protein) |  |  |  | |  |  |  |
| TBS | 38 | 196.1 (0.0, 4273.3) | 148 | | 215.5 (0.0, 1539.5) | 141 | 552.2 (0.0, 2351.2) |
| TBSX | 42 | 142.5 (0.0, 2227.4) | 150 | | 387.3 (0.0, 2596.4) | 143 | 960.7 (0.0, 2904.5) |
| FA | 45 | 105217.4 (0.0, 2448387.1) | 152 | | 203294.1 (0.0, 2090000.0) | 151 | 735042.7 (0.0, 3037837.8) |
| apoE (ng/mg protein) |  |  |  | |  |  |  |
| TBS | 42 | 555.5 (203.8, 1325.3) | 154 | | 488.8 (153.1, 1191.8) | 149 | 443.5 (142.1, 1192.5) |
| TBSX | 43 | 113.2 (46.1, 246.5) | 151 | | 118.4 (41.0, 566.1) | 145 | 145.0 (39.8, 405.1) |
| FA | 44 | 31.1 (0.0, 1731.0) | 153 | | 33.7 (0.0, 782.0) | 149 | 128.8 (0.0, 933.8) |
| tTau (ng/mg protein) |  |  |  | |  |  |  |
| TBS | 40 | 1625.3 (0.0, 3834.9) | 136 | | 1884.3 (0.0, 4873.4) | 142 | 1594.3 (0.0, 5183.2) |
| TBSX | 43 | 185.5 (72.8, 461.5) | 151 | | 195.8 (37.4, 1459.4) | 145 | 156.9 (33.0, 856.8) |
| FA | 45 | 136.2 (40.7, 319.9) | 156 | | 116.1 (45.6, 394.5) | 152 | 99.9 (23.1, 323.0) |
| pTau181 (ng/mg protein) |  |  |  | |  |  |  |
| TBS | 42 | 7.8 (0.0, 20.5) | 152 | | 8.7 (0.0, 38.3) | 145 | 6.6 (0.0, 30.4) |
| TBSX | 43 | 3.4 (0.6, 10.8) | 151 | | 3.4 (0.6, 25.4) | 144 | 3.4 (0.0, 15.4) |
| FA | 44 | 1.1 (0.0, 81.2) | 148 | | 1.5 (0.0, 115.0) | 148 | 5.9 (0.0, 184.7) |

**Table S4: Associations of MMSE score with AD-related molecule levels**

|  | Unadjusted analysis | | Adjusting for age and sex | |
| --- | --- | --- | --- | --- |
| Variable | β (95% CI) | P-value | β (95% CI) | P-value |
| TBS fraction |  |  |  |  |
| Aβ40 | 0.004 (-0.051, 0.058) | 0.89 | 0.001 (-0.051, 0.053) | 0.96 |
| Aβ42 | -0.039 (-0.072, -0.006) | 0.022 | -0.029 (-0.062, 0.004) | 0.086 |
| apoE | 0.023 (-0.068, 0.115) | 0.62 | -0.022 (-0.113, 0.069) | 0.64 |
| tTau | 0.000 (-0.007, 0.007) | 0.98 | 0.000 (-0.006, 0.007) | 0.92 |
| pTau181 | 0.151 (-0.075, 0.377) | 0.19 | 0.096 (-0.124, 0.316) | 0.39 |
| TBSX fraction |  |  |  |  |
| Aβ40 | -0.017 (-0.082, 0.047) | 0.59 | -0.020 (-0.082, 0.043) | 0.54 |
| Aβ42 | -0.033 (-0.065, -0.001) | 0.042 | -0.023 (-0.056, 0.009) | 0.15 |
| apoE | 0.230 (0.021, 0.439) | 0.031 | 0.188 (-0.018, 0.395) | 0.074 |
| tTau | 0.211 (-0.016, 0.438) | 0.068 | 0.292 (0.072, 0.513) | 0.010 |
| pTau181 | 0.177 (-0.061, 0.414) | 0.14 | 0.217 (-0.017, 0.451) | 0.068 |
| FA fraction |  |  |  |  |
| Aβ40 | -0.009 (-0.018, -0.001) | 0.032 | -0.008 (-0.017, 0.000) | 0.049 |
| Aβ42 | **-0.005 (-0.008, -0.002)** | **0.002** | -0.004 (-0.007, -0.001) | 0.012 |
| apoE | -0.032 (-0.109, 0.046) | 0.42 | -0.025 (-0.100, 0.050) | 0.51 |
| tTau | 0.255 (0.047, 0.464) | 0.017 | **0.274 (0.075, 0.474)** | **0.007** |
| pTau181 | -0.209 (-0.392, -0.025) | 0.026 | -0.181 (-0.359, -0.003) | 0.047 |
| β=regression coefficient; CI=confidence interval. β values, 95% CIs, and p-values result from linear regression models. β values are interpreted as the change in mean AD-related molecule on the square root (tTau-TBS), cube root (apoE-TBSX, tTau-TBSX, tTau-FA), or natural logarithm scale (Aβ40-TBS, Aβ40-TBSX, Aβ40-FA, Aβ42-TBS, Aβ42-TBSX, Aβ42-FA, apoE-TBS, apoE-FA, pTau181-TBS, pTau181-TBSX, pTau181-FA) corresponding to each 5-unit increase in MMSE score. P-values <0.01 were considered as statistically significant after applying a Bonferroni correction for multiple testing separately for each fraction; significant findings are shown in bold. | | | | |

**Table S5:** **Associations of neuropathological measures with AD-related molecules (TBS)**

|  |  | Unadjusted analysis | | Adjusting for age and sex | | Full multivariable analysis | |
| --- | --- | --- | --- | --- | --- | --- | --- |
| Variable | N | β (95% CI) | P-value | β (95% CI) | P-value | β (95% CI) | P-value |
| Association with Aβ40-TBS |  |  |  |  |  |  |  |
| Age | 306 | -0.15 (-0.48, 0.19) | 0.39 | -0.22 (-0.56, 0.12) | 0.20 | -0.03 (-0.37, 0.31) | 0.86 |
| Sex | 306 | -0.45 (-0.97, 0.07) | 0.092 | -0.53 (-1.06, 0.01) | 0.054 | -0.64 (-1.16, -0.12) | 0.017 |
| *APOE4* | 306 | 0.70 (0.18, 1.23) | 0.009 | 0.71 (0.17, 1.25) | 0.011 | 0.45 (-0.14, 1.04) | 0.13 |
| VaD | 306 | -0.18 (-0.73, 0.38) | 0.53 | -0.10 (-0.66, 0.46) | 0.73 | -0.05 (-0.60, 0.49) | 0.84 |
| CAA | 306 | **2.18 (1.12, 3.25)** | **<0.001** | **2.31 (1.25, 3.37)** | **<0.001** | **2.30 (1.24, 3.36)** | **<0.001** |
| Neuritic plaque score | 306 | **0.36 (0.13, 0.58)** | **0.002** | **0.33 (0.10, 0.56)** | **0.004** | 0.13 (-0.17, 0.43) | 0.39 |
| TDP-43 | 306 | 0.34 (-0.66, 1.33) | 0.51 | 0.21 (-0.79, 1.22) | 0.68 | -0.15 (-1.14, 0.83) | 0.76 |
| Synuclein | 306 | -0.08 (-0.71, 0.55) | 0.81 | -0.10 (-0.74, 0.54) | 0.75 | -0.41 (-1.04, 0.22) | 0.20 |
| AD-tau | 301 | **0.79 (0.26, 1.31)** | **0.004** | 0.74 (0.20, 1.27) | 0.007 | 0.42 (-0.28, 1.11) | 0.24 |
| AGD-tau | 306 | -0.52 (-1.27, 0.23) | 0.17 | -0.52 (-1.28, 0.23) | 0.17 | -0.35 (-1.09, 0.39) | 0.35 |
| Association with Aβ42-TBS |  |  |  |  |  |  |  |
| Age | 327 | -0.37 (-0.79, 0.05) | 0.081 | -0.34 (-0.77, 0.09) | 0.12 | 0.23 (-0.14, 0.60) | 0.23 |
| Sex | 327 | 0.33 (-0.34, 0.99) | 0.34 | 0.19 (-0.50, 0.88) | 0.59 | 0.41 (-0.16, 0.98) | 0.16 |
| *APOE4* | 327 | **2.22 (1.59, 2.85)** | **<0.001** | **2.23 (1.57, 2.89)** | **<0.001** | 0.88 (0.23, 1.52) | 0.008 |
| VaD | 327 | -0.12 (-0.82, 0.59) | 0.74 | -0.02 (-0.73, 0.69) | 0.96 | 0.21 (-0.39, 0.81) | 0.49 |
| CAA | 327 | 0.96 (-0.43, 2.35) | 0.17 | 0.88 (-0.52, 2.28) | 0.22 | 0.38 (-0.79, 1.55) | 0.52 |
| Neuritic plaque score | 327 | **1.51 (1.27, 1.74)** | **<0.001** | **1.53 (1.29, 1.77)** | **<0.001** | **1.23 (0.91, 1.56)** | **<0.001** |
| TDP-43 | 327 | 0.48 (-0.76, 1.71) | 0.45 | 0.55 (-0.69, 1.79) | 0.39 | -0.50 (-1.54, 0.54) | 0.34 |
| Synuclein | 327 | 0.54 (-0.24, 1.33) | 0.17 | 0.41 (-0.40, 1.21) | 0.32 | -0.57 (-1.24, 0.11) | 0.100 |
| AD-tau | 321 | **2.70 (2.09, 3.32)** | **<0.001** | **2.68 (2.06, 3.31)** | **<0.001** | 0.42 (-0.33, 1.17) | 0.27 |
| AGD-tau | 327 | **-1.70 (-2.64, -0.75)** | **<0.001** | **-1.60 (-2.56, -0.65)** | **0.001** | -0.43 (-1.25, 0.38) | 0.29 |
| Association with apoE-TBS |  |  |  |  |  |  |  |
| Age | 345 | -0.19 (-0.32, -0.06) | 0.005 | -0.16 (-0.30, -0.03) | 0.018 | **-0.23 (-0.37, -0.09)** | **0.001** |
| Sex | 345 | 0.24 (0.03, 0.45) | 0.026 | 0.18 (-0.04, 0.40) | 0.11 | 0.17 (-0.04, 0.39) | 0.11 |
| *APOE4* | 345 | **-0.31 (-0.53, -0.10)** | **0.004** | **-0.43 (-0.65, -0.22)** | **<0.001** | **-0.41 (-0.63, -0.19)** | **<0.001** |
| VaD | 345 | 0.12 (-0.10, 0.35) | 0.29 | 0.16 (-0.06, 0.39) | 0.15 | 0.12 (-0.11, 0.34) | 0.31 |
| CAA | 345 | 0.20 (-0.23, 0.63) | 0.36 | 0.13 (-0.29, 0.56) | 0.54 | 0.16 (-0.26, 0.58) | 0.46 |
| Neuritic plaque score | 345 | -0.01 (-0.10, 0.09) | 0.88 | -0.03 (-0.12, 0.07) | 0.57 | 0.06 (-0.04, 0.16) | 0.24 |
| TDP-43 | 345 | -0.46 (-0.86, -0.06) | 0.025 | -0.42 (-0.82, -0.02) | 0.041 | -0.29 (-0.69, 0.11) | 0.15 |
| Synuclein | 345 | -0.02 (-0.27, 0.23) | 0.87 | -0.11 (-0.36, 0.14) | 0.40 | -0.03 (-0.28, 0.22) | 0.82 |
| AD-tau | 339 | -0.16 (-0.38, 0.06) | 0.15 | -0.22 (-0.43, -0.00) | 0.050 | -0.05 (-0.29, 0.18) | 0.65 |
| AGD-tau | 345 | -0.11 (-0.42, 0.19) | 0.46 | -0.04 (-0.34, 0.27) | 0.81 | -0.09 (-0.39, 0.21) | 0.57 |
| Association with tTau-TBS |  |  |  |  |  |  |  |
| Age | 318 | 1.57 (-0.38, 3.51) | 0.11 | 1.49 (-0.52, 3.50) | 0.14 | 0.72 (-1.32, 2.77) | 0.49 |
| Sex | 318 | -1.10 (-4.26, 2.07) | 0.50 | -0.49 (-3.76, 2.77) | 0.77 | -0.06 (-3.33, 3.21) | 0.97 |
| *APOE4* | 318 | -1.57 (-4.75, 1.61) | 0.33 | -0.89 (-4.22, 2.43) | 0.60 | 0.74 (-2.89, 4.37) | 0.69 |
| VaD | 318 | -1.94 (-5.25, 1.38) | 0.25 | -2.50 (-5.89, 0.89) | 0.15 | -2.13 (-5.55, 1.29) | 0.22 |
| CAA | 318 | -5.38 (-11.59, 0.83) | 0.089 | -5.10 (-11.37, 1.17) | 0.11 | -5.45 (-11.65, 0.76) | 0.085 |
| Neuritic plaque score | 318 | -1.15 (-2.50, 0.20) | 0.094 | -0.99 (-2.37, 0.38) | 0.16 | -0.41 (-2.27, 1.46) | 0.67 |
| TDP-43 | 318 | -0.99 (-6.99, 5.00) | 0.74 | -1.24 (-7.25, 4.77) | 0.68 | -0.97 (-7.01, 5.07) | 0.75 |
| Synuclein | 318 | -0.78 (-4.49, 2.93) | 0.68 | -0.14 (-3.94, 3.66) | 0.94 | 0.06 (-3.78, 3.90) | 0.97 |
| AD-tau | 312 | -3.50 (-6.69, -0.30) | 0.032 | -3.24 (-6.49, 0.02) | 0.051 | -2.62 (-5.95, 0.71) | 0.12 |
| AGD-tau | 318 | 4.90 (0.51, 9.29) | 0.029 | 4.47 (0.01, 8.93) | 0.049 | 3.71 (-0.85, 8.28) | 0.11 |
| Association with pTau181-TBS |  |  |  |  |  |  |  |
| Age | 339 | 0.06 (-0.01, 0.13) | 0.080 | 0.08 (0.01, 0.14) | 0.028 | 0.03 (-0.03, 0.10) | 0.32 |
| Sex | 339 | 0.09 (-0.02, 0.19) | 0.12 | 0.12 (0.01, 0.23) | 0.040 | 0.09 (-0.02, 0.20) | 0.095 |
| *APOE4* | 339 | **-0.16 (-0.26, -0.05)** | **0.004** | -0.14 (-0.25, -0.03) | 0.012 | -0.03 (-0.15, 0.09) | 0.61 |
| VaD | 339 | 0.05 (-0.06, 0.16) | 0.38 | 0.02 (-0.09, 0.14) | 0.68 | 0.00 (-0.11, 0.11) | 0.99 |
| CAA | 339 | 0.15 (-0.06, 0.37) | 0.16 | 0.13 (-0.08, 0.35) | 0.22 | 0.17 (-0.04, 0.38) | 0.11 |
| Neuritic plaque score | 339 | **-0.11 (-0.15, -0.06)** | **<0.001** | **-0.10 (-0.15, -0.06)** | **<0.001** | -0.05 (-0.11, 0.01) | 0.095 |
| TDP-43 | 339 | -0.09 (-0.29, 0.11) | 0.39 | -0.07 (-0.27, 0.13) | 0.48 | 0.00 (-0.19, 0.20) | 0.99 |
| Synuclein | 339 | **-0.18 (-0.31, -0.06)** | **0.004** | -0.17 (-0.30, -0.05) | 0.008 | -0.10 (-0.22, 0.03) | 0.13 |
| AD-tau | 333 | **-0.25 (-0.36, -0.15)** | **<0.001** | **-0.24 (-0.34, -0.13)** | **<0.001** | -0.14 (-0.28, 0.01) | 0.061 |
| AGD-tau | 339 | 0.11 (-0.04, 0.26) | 0.15 | 0.11 (-0.05, 0.26) | 0.18 | 0.00 (-0.15, 0.15) | 0.97 |
| β=regression coefficient; CI=confidence interval. β values, 95% CIs, and p-values result from linear regression models. β values are interpreted as the change in mean AD-related molecule on the square root (tTau-TBS), or natural logarithm scale (Aβ40-TBS, Aβ42-TBS, apoE-TBS, pTau181-TBS) corresponding to each 10-year increase in age, male sex, presence of *APOE4*, presence of VaD, presence of CAA, 1 unit increase in neuritic plaque score, presence of TDP-43 pathology, presence of synucleinopathy, presence of AD-tau or presence of AGD-tau. Full multivariable models were adjusted for age, sex, Braak stage, and all other variables with an association p-value <0.05 in the unadjusted analysis for the given AD-related molecule. P-values <0.005 were considered as statistically significant after applying a Bonferroni correction for multiple testing separately for each AD-related molecule; significant findings are shown in bold. | | | | | | | |

**Table S6: Associations of neuropathological measures with AD-related molecules (TBSX)**

|  |  | Unadjusted analysis | | Adjusting for age and sex | | Full multivariable analysis | |
| --- | --- | --- | --- | --- | --- | --- | --- |
| Variable | N | β (95% CI) | P-value | β (95% CI) | P-value | β (95% CI) | P-value |
| Association with Aβ40-TBSX |  |  |  |  |  |  |  |
| Age | 332 | -0.34 (-0.61, -0.07) | 0.013 | -0.36 (-0.64, -0.08) | 0.013 | -0.14 (-0.43, 0.14) | 0.32 |
| Sex | 332 | 0.05 (-0.39, 0.49) | 0.82 | -0.09 (-0.54, 0.37) | 0.70 | -0.19 (-0.63, 0.26) | 0.40 |
| *APOE4* | 332 | **1.11 (0.67, 1.54)** | **<0.001** | **1.03 (0.58, 1.48)** | **<0.001** | **0.79 (0.29, 1.29)** | **0.002** |
| VaD | 332 | 0.30 (-0.16, 0.77) | 0.20 | 0.43 (-0.04, 0.90) | 0.072 | 0.48 (0.03, 0.94) | 0.039 |
| CAA | 332 | **1.52 (0.66, 2.38)** | **<0.001** | **1.53 (0.66, 2.39)** | **<0.001** | **1.41 (0.57, 2.26)** | **0.001** |
| Neuritic plaque score | 332 | **0.36 (0.17, 0.54)** | **<0.001** | **0.32 (0.13, 0.51)** | **0.001** | -0.05 (-0.31, 0.22) | 0.73 |
| TDP-43 | 332 | 0.28 (-0.56, 1.12) | 0.51 | 0.28 (-0.56, 1.11) | 0.52 | -0.13 (-0.95, 0.69) | 0.76 |
| Synuclein | 332 | 0.68 (0.17, 1.20) | 0.010 | 0.59 (0.07, 1.12) | 0.028 | 0.25 (-0.28, 0.77) | 0.35 |
| AD-tau | 327 | **0.92 (0.48, 1.36)** | **<0.001** | **0.83 (0.38, 1.28)** | **<0.001** | 0.52 (-0.07, 1.11) | 0.085 |
| AGD-tau | 332 | -0.74 (-1.37, -0.11) | 0.021 | -0.65 (-1.28, -0.02) | 0.044 | -0.40 (-1.02, 0.23) | 0.21 |
| Association with Aβ42-TBSX |  |  |  |  |  |  |  |
| Age | 335 | -0.35 (-0.76, 0.05) | 0.087 | -0.35 (-0.77, 0.07) | 0.10 | 0.32 (0.00, 0.64) | 0.048 |
| Sex | 335 | 0.16 (-0.50, 0.81) | 0.64 | 0.02 (-0.65, 0.69) | 0.95 | 0.28 (-0.21, 0.77) | 0.27 |
| *APOE4* | 335 | **2.68 (2.08, 3.27)** | **<0.001** | **2.75 (2.12, 3.37)** | **<0.001** | **1.15 (0.59, 1.72)** | **<0.001** |
| VaD | 335 | -0.35 (-1.03, 0.33) | 0.31 | -0.26 (-0.96, 0.43) | 0.46 | 0.01 (-0.51, 0.52) | 0.97 |
| CAA | 335 | 0.65 (-0.62, 1.91) | 0.31 | 0.60 (-0.68, 1.88) | 0.36 | -0.10 (-1.04, 0.83) | 0.83 |
| Neuritic plaque score | 335 | **1.75 (1.54, 1.96)** | **<0.001** | **1.77 (1.56, 1.98)** | **<0.001** | **1.33 (1.04, 1.61)** | **<0.001** |
| TDP-43 | 335 | 1.28 (0.07, 2.49) | 0.039 | 1.30 (0.08, 2.52) | 0.036 | -0.05 (-0.95, 0.85) | 0.91 |
| Synuclein | 335 | **1.12 (0.36, 1.88)** | **0.004** | 1.03 (0.26, 1.81) | 0.009 | -0.15 (-0.74, 0.43) | 0.60 |
| AD-tau | 330 | **3.38 (2.82, 3.93)** | **<0.001** | **3.39 (2.82, 3.95)** | **<0.001** | 0.90 (0.24, 1.55) | 0.007 |
| AGD-tau | 335 | **-1.78 (-2.70, -0.86)** | **<0.001** | **-1.69 (-2.63, -0.75)** | **<0.001** | -0.19 (-0.90, 0.52) | 0.60 |
| Association with apoE-TBSX |  |  |  |  |  |  |  |
| Age | 339 | **-0.15 (-0.20, -0.09)** | **<0.001** | **-0.13 (-0.19, -0.07)** | **<0.001** | **-0.09 (-0.15, -0.03)** | **0.003** |
| Sex | 339 | 0.13 (0.04, 0.23) | 0.006 | 0.08 (-0.01, 0.18) | 0.088 | 0.06 (-0.04, 0.16) | 0.22 |
| *APOE4* | 339 | **0.20 (0.11, 0.30)** | **<0.001** | **0.14 (0.05, 0.24)** | **0.004** | 0.10 (-0.00, 0.21) | 0.055 |
| VaD | 339 | -0.05 (-0.16, 0.05) | 0.29 | -0.02 (-0.12, 0.07) | 0.62 | -0.03 (-0.13, 0.07) | 0.60 |
| CAA | 339 | 0.20 (0.01, 0.39) | 0.040 | 0.16 (-0.02, 0.34) | 0.090 | 0.13 (-0.06, 0.31) | 0.17 |
| Neuritic plaque score | 339 | **0.07 (0.03, 0.11)** | **0.001** | 0.05 (0.01, 0.10) | 0.009 | 0.01 (-0.03, 0.06) | 0.56 |
| TDP-43 | 339 | -0.05 (-0.24, 0.13) | 0.56 | -0.04 (-0.21, 0.14) | 0.68 | -0.09 (-0.26, 0.09) | 0.33 |
| Synuclein | 339 | **0.19 (0.08, 0.30)** | **<0.001** | 0.14 (0.03, 0.25) | 0.016 | 0.10 (-0.01, 0.21) | 0.088 |
| AD-tau | 334 | 0.10 (-0.00, 0.19) | 0.058 | 0.06 (-0.04, 0.15) | 0.26 | -0.07 (-0.20, 0.06) | 0.27 |
| AGD-tau | 339 | **-0.24 (-0.38, -0.11)** | **<0.001** | -0.19 (-0.32, -0.06) | 0.005 | -0.16 (-0.30, -0.03) | 0.019 |
| Association with tTau-TBSX |  |  |  |  |  |  |  |
| Age | 339 | **0.09 (0.03, 0.16)** | **0.004** | **0.12 (0.05, 0.18)** | **<0.001** | 0.07 (0.01, 0.14) | 0.024 |
| Sex | 339 | 0.11 (0.01, 0.22) | 0.031 | **0.16 (0.05, 0.26)** | **0.003** | 0.13 (0.03, 0.23) | 0.010 |
| *APOE4* | 339 | **-0.20 (-0.30, -0.10)** | **<0.001** | **-0.17 (-0.28, -0.07)** | **0.001** | -0.03 (-0.15, 0.08) | 0.55 |
| VaD | 339 | 0.00 (-0.11, 0.11) | 0.98 | -0.04 (-0.15, 0.07) | 0.44 | -0.05 (-0.15, 0.05) | 0.34 |
| CAA | 339 | 0.11 (-0.10, 0.31) | 0.31 | 0.08 (-0.12, 0.28) | 0.43 | 0.10 (-0.09, 0.29) | 0.29 |
| Neuritic plaque score | 339 | **-0.13 (-0.17, -0.09)** | **<0.001** | **-0.12 (-0.16, -0.07)** | **<0.001** | -0.04 (-0.10, 0.01) | 0.14 |
| TDP-43 | 339 | -0.17 (-0.37, 0.02) | 0.083 | -0.15 (-0.34, 0.04) | 0.12 | -0.06 (-0.25, 0.12) | 0.52 |
| Synuclein | 339 | -0.09 (-0.22, 0.03) | 0.14 | -0.07 (-0.20, 0.05) | 0.23 | 0.01 (-0.11, 0.13) | 0.84 |
| AD-tau | 334 | **-0.34 (-0.44, -0.24)** | **<0.001** | **-0.31 (-0.41, -0.21)** | **<0.001** | **-0.23 (-0.37, -0.10)** | **<0.001** |
| AGD-tau | 339 | 0.07 (-0.08, 0.22) | 0.34 | 0.06 (-0.09, 0.21) | 0.42 | -0.06 (-0.20, 0.08) | 0.42 |
| Association with pTau181-TBSX |  |  |  |  |  |  |  |
| Age | 338 | 0.01 (-0.04, 0.06) | 0.70 | 0.03 (-0.02, 0.08) | 0.25 | 0.02 (-0.03, 0.07) | 0.46 |
| Sex | 338 | **0.13 (0.05, 0.20)** | **0.001** | **0.14 (0.06, 0.21)** | **<0.001** | **0.13 (0.05, 0.20)** | **0.002** |
| *APOE4* | 338 | -0.04 (-0.12, 0.03) | 0.27 | -0.05 (-0.13, 0.03) | 0.26 | -0.04 (-0.12, 0.04) | 0.35 |
| VaD | 338 | 0.11 (0.03, 0.19) | 0.009 | 0.09 (0.01, 0.17) | 0.022 | 0.09 (0.01, 0.17) | 0.022 |
| CAA | 338 | 0.13 (-0.02, 0.28) | 0.079 | 0.10 (-0.05, 0.25) | 0.17 | 0.09 (-0.05, 0.24) | 0.21 |
| Neuritic plaque score | 338 | -0.03 (-0.06, 0.00) | 0.072 | -0.03 (-0.06, 0.01) | 0.13 | -0.02 (-0.06, 0.01) | 0.16 |
| TDP-43 | 338 | -0.01 (-0.15, 0.14) | 0.90 | 0.01 (-0.13, 0.16) | 0.87 | 0.01 (-0.13, 0.15) | 0.91 |
| Synuclein | 338 | -0.04 (-0.13, 0.05) | 0.39 | -0.05 (-0.14, 0.04) | 0.28 | -0.04 (-0.13, 0.05) | 0.38 |
| AD-tau | 333 | -0.05 (-0.13, 0.03) | 0.22 | -0.04 (-0.12, 0.04) | 0.30 | -0.04 (-0.12, 0.04) | 0.30 |
| AGD-tau | 338 | 0.06 (-0.05, 0.17) | 0.28 | 0.07 (-0.04, 0.18) | 0.19 | 0.09 (-0.02, 0.20) | 0.10 |
| β=regression coefficient; CI=confidence interval. β values, 95% CIs, and p-values result from linear regression models. β values are interpreted as the change in mean AD-related molecule on the cube root (apoE-TBSX, tTau-TBSX), or natural logarithm scale (Aβ40-TBSX, Aβ42-TBSX, pTau181-TBSX) corresponding to each 10-year increase in age, male sex, presence of *APOE4*, presence of VaD, presence of CAA, 1 unit increase in neuritic plaque score, presence of TDP-43 pathology, presence of synucleinopathy, presence of AD-tau or presence of AGD-tau. Full multivariable models were adjusted for age, sex, and all other variables with an association p-value <0.05 in the unadjusted analysis for the given AD-related molecule. P-values <0.005 were considered as statistically significant after applying a Bonferroni correction for multiple testing separately for each AD-related molecule; significant findings are shown in bold. | | | | | | | |

**Table S7: Subject characteristics according to combination of AD-tau and AGD-tau pathology**

|  | No tau pathology  (N=114) | | AD-tau pathology only (N=182) | | AGD-tau pathology only (N=36) | | AD-tau and AGD-tau pathology (N=15) | |  |
| --- | --- | --- | --- | --- | --- | --- | --- | --- | --- |
| Variable | N | Median (minimum, maximum) or No. (%) of patients | N | Median (minimum, maximum) or No. (%) of patients | N | Median (minimum, maximum) or No. (%) of patients | N | Median (minimum, maximum) or No. (%) of patients | P-value |
| Age at death (years) | 114 | 88 (61, 101) | 182 | 85 (54, 103) | 36 | 88.5 (77, 96) | 15 | 89 (70, 100) | 0.002 |
| Sex (Male) | 114 | 61 (53.5%) | 182 | 94 (51.6%) | 36 | 13 (36.1%) | 15 | 5 (33.3%) | 0.16 |
| MMSE score | 70 | 27 (22, 30) | 36 | 25 (17, 29) | 19 | 27 (22, 29) | 8 | 27 (21, 29) | <0.001 |
| *APOE* genotype | 114 |  | 182 |  | 36 |  | 15 |  | <0.001 |
| ε2/ε2 |  | 0 (0.0%) |  | 0 (0.0%) |  | 0 (0.0%) |  | 1 (6.7%) |  |
| ε2/ε3 |  | 21 (18.4%) |  | 13 (7.1%) |  | 8 (22.2%) |  | 2 (13.3%) |  |
| ε3/ε3 |  | 73 (64.0%) |  | 54 (29.7%) |  | 20 (55.6%) |  | 7 (46.7%) |  |
| ε3/ε4 |  | 19 (16.7%) |  | 77 (42.3%) |  | 6 (16.7%) |  | 5 (33.3%) |  |
| ε4/ε4 |  | 1 (0.9%) |  | 38 (20.9%) |  | 2 (5.6%) |  | 0 (0.0%) |  |
| Braak stage | 114 |  | 182 |  | 36 |  | 15 |  | <0.001 |
| 0 |  | 5 (4.4%) |  | 0 (0.0%) |  | 1 (2.8%) |  | 0 (0.0%) |  |
| 1 |  | 24 (21.1%) |  | 0 (0.0%) |  | 4 (11.1%) |  | 0 (0.0%) |  |
| 2 |  | 45 (39.5%) |  | 0 (0.0%) |  | 16 (44.4%) |  | 0 (0.0%) |  |
| 3 |  | 40 (35.1%) |  | 0 (0.0%) |  | 15 (41.7%) |  | 0 (0.0%) |  |
| 4 |  | 0 (0.0%) |  | 54 (29.7%) |  | 0 (0.0%) |  | 10 (66.7%) |  |
| 5 |  | 0 (0.0%) |  | 58 (31.9%) |  | 0 (0.0%) |  | 5 (33.3%) |  |
| 6 |  | 0 (0.0%) |  | 70 (38.5%) |  | 0 (0.0%) |  | 0 (0.0%) |  |
| Thal phase | 84 |  | 75 |  | 22 |  | 11 |  | <0.001 |
| 0 |  | 25 (29.8%) |  | 2 (2.7%) |  | 7 (31.8%) |  | 2 (18.2%) |  |
| 1 |  | 21 (25.0%) |  | 7 (9.3%) |  | 5 (22.7%) |  | 1 (9.1%) |  |
| 2 |  | 9 (10.7%) |  | 7 (9.3%) |  | 3 (13.6%) |  | 0 (0.0%) |  |
| 3 |  | 21 (25.0%) |  | 16 (21.3%) |  | 7 (31.8%) |  | 2 (18.2%) |  |
| 4 |  | 4 (4.8%) |  | 7 (9.3%) |  | 0 (0.0%) |  | 2 (18.2%) |  |
| 5 |  | 4 (4.8%) |  | 36 (48.0%) |  | 0 (0.0%) |  | 4 (36.4%) |  |
| VaD | 114 | 47 (41.2%) | 182 | 64 (35.2%) | 36 | 9 (25.0%) | 15 | 2 (13.3%) | 0.091 |
| CAA | 114 | 9 (7.9%) | 182 | 12 (6.6%) | 36 | 2 (5.6%) | 15 | 1 (6.7%) | 0.96 |
| Neuritic plaque score | 114 |  | 182 |  | 36 |  | 15 |  | <0.001 |
| 0 |  | 54 (47.4%) |  | 5 (2.7%) |  | 17 (47.2%) |  | 3 (20.0%) |  |
| 1 |  | 25 (21.9%) |  | 21 (11.5%) |  | 15 (41.7%) |  | 3 (20.0%) |  |
| 2 |  | 32 (28.1%) |  | 45 (24.7%) |  | 4 (11.1%) |  | 3 (20.0%) |  |
| 3 |  | 3 (2.6%) |  | 111 (61.0%) |  | 0 (0.0%) |  | 6 (40.0%) |  |
| TDP-43 | 114 | 1 (0.9%) | 182 | 20 (11.0%) | 36 | 4 (11.1%) | 15 | 2 (13.3%) | 0.004 |
| Synuclein | 114 | 20 (17.5%) | 182 | 55 (30.2%) | 36 | 5 (13.9%) | 15 | 3 (20.0%) | 0.042 |
| P-values result from a Kruskal-Wallis rank sum test (continuous and ordinal variables) or Fisher’s exact test (categorical variables). | | | | | | | | | |

**Table S8: Interactions of AGD-tau with neuropathological measures regarding associations with AD-related molecules**

|  | No AGD-tau pathology (N=302) | | AGD-tau pathology (N=51) | |  |
| --- | --- | --- | --- | --- | --- |
| Variable | β (95% CI) | P-value | β (95% CI) | P-value | Interaction p-value |
| Association with Aβ40-FA |  |  |  |  |  |
| Age | -2.45 (-5.24, 0.33) | 0.084 | 1.58 (-1.99, 5.14) | 0.38 | 0.41 |
| Sex | 0.06 (-4.46, 4.59) | 0.98 | 1.60 (-2.62, 5.83) | 0.45 | 0.84 |
| *APOE4* | **15.19 (10.99, 19.40)** | **<0.001** | 0.89 (-3.75, 5.54) | 0.70 | 0.011 |
| VaD | -2.03 (-6.68, 2.61) | 0.39 | 2.43 (-2.31, 7.17) | 0.31 | 0.47 |
| CAA | **25.95 (17.98, 33.93)** | **<0.001** | 1.60 (-6.75, 9.95) | 0.70 | 0.027 |
| Neuritic plaque score | **6.55 (4.75, 8.36)** | **<0.001** | 0.12 (-1.91, 2.14) | 0.91 | 0.011 |
| TDP-43 | 1.18 (-7.66, 10.02) | 0.79 | 2.11 (-4.21, 8.43) | 0.50 | 0.92 |
| Synuclein | 5.17 (0.06, 10.27) | 0.047 | -0.68 (-6.09, 4.73) | 0.80 | 0.40 |
| AD-tau | **12.97 (8.55, 17.40)** | **<0.001** | 2.91 (-1.45, 7.26) | 0.19 | 0.076 |
| Association with Aβ42-FA |  |  |  |  |  |
| Age | -3.52 (-8.05, 1.01) | 0.13 | 2.27 (-12.95, 17.50) | 0.77 | 0.48 |
| Sex | -1.32 (-8.80, 6.16) | 0.73 | -4.13 (-21.67, 13.41) | 0.64 | 0.76 |
| *APOE4* | **23.65 (16.57, 30.72)** | **<0.001** | **27.13 (8.84, 45.41)** | **0.004** | 0.89 |
| VaD | -1.60 (-9.29, 6.08) | 0.68 | 0.56 (-20.05, 21.17) | 0.96 | 0.85 |
| CAA | **21.19 (6.77, 35.62)** | **0.004** | 11.26 (-24.73, 47.26) | 0.53 | 0.65 |
| Neuritic plaque score | **17.83 (15.29, 20.37)** | **<0.001** | **19.39 (12.89, 25.89)** | **<0.001** | 0.70 |
| TDP-43 | 11.56 (-2.53, 25.66) | 0.11 | 19.61 (-7.20, 46.42) | 0.15 | 0.58 |
| Synuclein | 10.27 (1.84, 18.71) | 0.017 | -0.05 (-23.41, 23.32) | 1.00 | 0.39 |
| AD-tau | **34.95 (28.41, 41.48)** | **<0.001** | **28.34 (11.69, 44.99)** | **0.001** | 0.47 |
| Association with apoE-FA |  |  |  |  |  |
| Age | **-0.52 (-0.78, -0.25)** | **<0.001** | -0.07 (-0.79, 0.64) | 0.83 | 0.35 |
| Sex | 0.10 (-0.33, 0.53) | 0.65 | -0.02 (-0.84, 0.81) | 0.97 | 0.82 |
| *APOE4* | **1.27 (0.86, 1.69)** | **<0.001** | 0.94 (0.05, 1.83) | 0.040 | 0.44 |
| VaD | -0.37 (-0.81, 0.07) | 0.10 | 0.41 (-0.55, 1.37) | 0.39 | 0.22 |
| CAA | **1.41 (0.60, 2.23)** | **<0.001** | -0.53 (-2.21, 1.16) | 0.53 | 0.098 |
| Neuritic plaque score | **0.74 (0.57, 0.91)** | **<0.001** | 0.45 (0.06, 0.84) | 0.023 | 0.26 |
| TDP-43 | 0.63 (-0.21, 1.47) | 0.14 | 0.16 (-1.13, 1.45) | 0.80 | 0.68 |
| Synuclein | 0.40 (-0.09, 0.90) | 0.11 | 0.00 (-1.09, 1.10) | 1.00 | 0.54 |
| AD-tau | **1.05 (0.62, 1.48)** | **<0.001** | 0.11 (-0.77, 0.98) | 0.81 | 0.11 |
| Association with tTau-FA |  |  |  |  |  |
| Age | 0.03 (-0.04, 0.09) | 0.44 | 0.01 (-0.22, 0.24) | 0.93 | 0.90 |
| Sex | -0.03 (-0.14, 0.08) | 0.63 | -0.02 (-0.29, 0.25) | 0.89 | 0.95 |
| *APOE4* | -0.15 (-0.26, -0.04) | 0.007 | -0.16 (-0.46, 0.14) | 0.28 | 0.98 |
| VaD | 0.01 (-0.11, 0.12) | 0.92 | 0.09 (-0.23, 0.40) | 0.58 | 0.64 |
| CAA | -0.03 (-0.25, 0.18) | 0.77 | -0.19 (-0.74, 0.36) | 0.49 | 0.59 |
| Neuritic plaque score | -0.05 (-0.09, 0.00) | 0.054 | 0.02 (-0.11, 0.15) | 0.80 | 0.38 |
| TDP-43 | -0.15 (-0.36, 0.06) | 0.16 | -0.14 (-0.55, 0.28) | 0.51 | 0.94 |
| Synuclein | -0.06 (-0.19, 0.06) | 0.34 | -0.35 (-0.69, -0.01) | 0.043 | 0.13 |
| AD-tau | -0.15 (-0.26, -0.04) | 0.007 | -0.07 (-0.35, 0.21) | 0.63 | 0.58 |
| Association with pTau181-FA |  |  |  |  |  |
| Age | **-0.40 (-0.56, -0.25)** | **<0.001** | -0.09 (-0.36, 0.17) | 0.47 | 0.27 |
| Sex | -0.13 (-0.39, 0.12) | 0.30 | -0.15 (-0.45, 0.16) | 0.34 | 0.94 |
| *APOE4* | **0.81 (0.58, 1.05)** | **<0.001** | -0.03 (-0.35, 0.30) | 0.88 | 0.008 |
| VaD | 0.00 (-0.26, 0.27) | 0.97 | 0.04 (-0.30, 0.38) | 0.81 | 0.88 |
| CAA | 0.44 (-0.04, 0.92) | 0.073 | 0.43 (-0.15, 1.00) | 0.14 | 0.94 |
| Neuritic plaque score | **0.52 (0.43, 0.61)** | **<0.001** | **0.20 (0.06, 0.33)** | **0.005** | 0.017 |
| TDP-43 | 0.48 (-0.00, 0.97) | 0.051 | 0.16 (-0.32, 0.64) | 0.50 | 0.63 |
| Synuclein | 0.21 (-0.08, 0.50) | 0.16 | 0.18 (-0.22, 0.59) | 0.37 | 0.80 |
| AD-tau | **1.06 (0.84, 1.29)** | **<0.001** | 0.33 (0.03, 0.63) | 0.032 | 0.013 |
| β=regression coefficient; CI=confidence interval. β values, 95% CIs, and p-values result from linear regression models that were adjusted for age and sex. β values are interpreted as the change in mean AD-related molecule on the cube root (Aβ40-FA, Aβ42-FA, apoE-FA, pTau181-FA) or natural logarithm scale (tTau-FA) corresponding to each 10-year increase in age, male sex, presence of *APOE4*, presence of VaD, presence of CAA, 1 unit increase in neuritic plaque score, presence of TDP-43 pathology, presence of synucleinopathy, or presence of AD-tau. For tests of interaction, models were additionally adjusted for AGD-tau and the interaction between AGD-tau and the given variable. P-values <0.0056 were considered as statistically significant after applying a Bonferroni correction for multiple testing separately for each AD-related molecule; significant interactions are shown in bold. | | | | | |

**Table S9: Interactions of AD-tau with neuropathological measures regarding associations with AD-related molecules**

|  | No AD-tau pathology (N=150) | | AD-tau pathology (N=197) | |  |
| --- | --- | --- | --- | --- | --- |
| Variable | β (95% CI) | P-value | β (95% CI) | P-value | Interaction p-value |
| Association with Aβ40-FA |  |  |  |  |  |
| Age | 1.00 (-2.17-4.18) | 0.53 | -1.74 (-5.23-1.74) | 0.32 | 0.26 |
| Sex | 0.34 (-3.87-4.54) | 0.87 | 1.90 (-4.01-7.81) | 0.53 | 0.55 |
| *APOE4* | 4.95 (-0.34-10.24) | 0.066 | **13.51 (7.79-19.24)** | **<0.001** | 0.037 |
| VaD | 1.16 (-3.20-5.53) | 0.60 | -2.46 (-8.58-3.67) | 0.43 | 0.33 |
| CAA | **20.52 (13.49-27.55)** | **<0.001** | **25.50 (14.80-36.21)** | **<0.001** | 0.44 |
| Neuritic plaque score | 2.26 (-0.14-4.66) | 0.065 | **6.83 (3.53-10.13)** | **<0.001** | 0.036 |
| TDP-43 | 0.00 (-11.42-11.41) | 1.00 | -2.93 (-12.14-6.28) | 0.53 | 0.67 |
| Synuclein | 1.24 (-4.44-6.91) | 0.67 | 4.53 (-1.84-10.90) | 0.16 | 0.41 |
| AGD-tau | -0.52 (-5.51-4.47) | 0.84 | -9.96 (-20.75-0.84) | 0.070 | 0.076 |
| Association with Aβ42-FA |  |  |  |  |  |
| Age | -0.13 (-7.73-7.47) | 0.97 | -0.18 (-4.34-3.98) | 0.93 | 0.96 |
| Sex | 2.68 (-7.23-12.59) | 0.59 | 0.75 (-6.50-8.00) | 0.84 | 0.75 |
| *APOE4* | **20.35 (8.19-32.51)** | **0.001** | 10.40 (3.04-17.76) | 0.006 | 0.13 |
| VaD | 0.54 (-9.66-10.74) | 0.92 | 2.62 (-4.99-10.23) | 0.50 | 0.76 |
| CAA | **26.67 (8.41-44.93)** | **0.004** | 17.81 (3.34-32.28) | 0.016 | 0.41 |
| Neuritic plaque score | **18.34 (13.55-23.13)** | **<0.001** | **12.09 (8.27-15.90)** | **<0.001** | 0.038 |
| TDP-43 | 2.98 (-24.37-30.34) | 0.83 | 2.69 (-8.36-13.74) | 0.63 | 1.00 |
| Synuclein | 6.10 (-7.24-19.44) | 0.37 | 4.85 (-3.04-12.74) | 0.23 | 0.84 |
| AGD-tau | -6.53 (-18.09-5.03) | 0.27 | -13.29 (-26.36, 0.22) | 0.046 | 0.47 |
| Association with apoE-FA |  |  |  |  |  |
| Age | -0.36 (-0.74-0.03) | 0.068 | -0.43 (-0.76, 0.11) | 0.008 | 0.70 |
| Sex | 0.06 (-0.44-0.56) | 0.81 | 0.25 (-0.30-0.81) | 0.37 | 0.58 |
| *APOE4* | 0.51 (-0.13-1.14) | 0.12 | **1.39 (0.85-1.93)** | **<0.001** | 0.044 |
| VaD | -0.29 (-0.81-0.22) | 0.27 | -0.14 (-0.72-0.44) | 0.64 | 0.71 |
| CAA | **1.51 (0.59-2.42)** | **0.001** | 0.93 (-0.14-2.01) | 0.089 | 0.48 |
| Neuritic plaque score | **0.55 (0.28-0.83)** | **<0.001** | **0.94 (0.65-1.23)** | **<0.001** | 0.068 |
| TDP-43 | -0.11 (-1.49-1.28) | 0.88 | 0.38 (-0.49-1.24) | 0.39 | 0.64 |
| Synuclein | 0.02 (-0.66-0.70) | 0.95 | 0.34 (-0.27-0.95) | 0.27 | 0.45 |
| AGD-tau | 0.09 (-0.50-0.68) | 0.76 | -0.81 (-1.81-0.20) | 0.12 | 0.11 |
| Association with tTau-FA |  |  |  |  |  |
| Age | 0.04 (-0.08-0.16) | 0.49 | 0.00 (-0.08-0.07) | 0.95 | 0.46 |
| Sex | -0.07 (-0.23-0.08) | 0.37 | -0.02 (-0.15-0.11) | 0.80 | 0.51 |
| *APOE4* | 0.00 (-0.20-0.20) | 1.00 | -0.17 (-0.30, 0.04) | 0.012 | 0.21 |
| VaD | 0.03 (-0.13-0.19) | 0.68 | 0.03 (-0.11-0.17) | 0.68 | 0.93 |
| CAA | -0.21 (-0.51-0.08) | 0.15 | 0.07 (-0.18-0.33) | 0.58 | 0.12 |
| Neuritic plaque score | -0.02 (-0.11-0.07) | 0.71 | 0.00 (-0.08-0.08) | 0.98 | 0.79 |
| TDP-43 | -0.20 (-0.63-0.23) | 0.36 | -0.08 (-0.28-0.12) | 0.43 | 0.66 |
| Synuclein | -0.03 (-0.25-0.18) | 0.76 | -0.12 (-0.26-0.02) | 0.11 | 0.60 |
| AGD-tau | -0.01 (-0.20-0.17) | 0.88 | 0.09 (-0.15-0.33) | 0.46 | 0.58 |
| Association with pTau181-FA |  |  |  |  |  |
| Age | 0.01 (-0.10-0.12) | 0.85 | **-0.43 (-0.61, 0.24)** | **<0.001** | **0.002** |
| Sex | 0.03 (-0.11-0.18) | 0.65 | -0.16 (-0.48-0.17) | 0.34 | 0.75 |
| *APOE4* | 0.17 (-0.01-0.36) | 0.068 | **0.50 (0.17-0.83)** | **0.003** | 0.086 |
| VaD | 0.02 (-0.14-0.17) | 0.83 | 0.10 (-0.24-0.44) | 0.55 | 0.89 |
| CAA | 0.05 (-0.22-0.33) | 0.69 | 0.83 (0.21-1.45) | 0.009 | 0.044 |
| Neuritic plaque score | 0.09 (0.01-0.17) | 0.028 | **0.54 (0.37-0.72)** | **<0.001** | **<0.001** |
| TDP-43 | 0.13 (-0.32-0.57) | 0.58 | 0.10 (-0.40-0.61) | 0.68 | 0.94 |
| Synuclein | 0.01 (-0.19-0.21) | 0.90 | 0.05 (-0.31-0.41) | 0.79 | 0.75 |
| AGD-tau | 0.03 (-0.15-0.20) | 0.76 | -0.68 (-1.27, 0.08) | 0.026 | 0.013 |
| β=regression coefficient; CI=confidence interval. β values, 95% CIs, and p-values result from linear regression models that were adjusted for age and sex. β values are interpreted as the change in mean AD-related molecule on the cube root (Aβ40-FA, Aβ42-FA, apoE-FA, pTau181-FA) or natural logarithm scale (tTau-FA) corresponding to each 10-year increase in age, male sex, presence of *APOE4*, presence of VaD, presence of CAA, 1 unit increase in neuritic plaque score, presence of TDP-43 pathology, presence of synucleinopathy, or presence of AGD-tau. For tests of interaction, models were additionally adjusted for AD-tau and the interaction between AD-tau and the given variable. P-values <0.0056 were considered as statistically significant after applying a Bonferroni correction for multiple testing separately for each AD-related molecule; significant associations are underlined, and significant interactions are shown in bold. | | | | | |
